# Looking deep inside tissue with photoacoustic molecular probes: a review

**DOI:** 10.1117/1.JBO.27.7.070901

**Published:** 2022-07-22

**Authors:** Xie Hui, Mohammad O. A. Malik, Manojit Pramanik

**Affiliations:** Nanyang Technological University, School of Chemical and Biomedical Engineering, Singapore

**Keywords:** photoacoustic imaging, molecular imaging, deep-tissue imaging, contrast agents, optical probe

## Abstract

**Significance:**

Deep tissue noninvasive high-resolution imaging with light is challenging due to the high degree of light absorption and scattering in biological tissue. Photoacoustic imaging (PAI) can overcome some of the challenges of pure optical or ultrasound imaging to provide high-resolution deep tissue imaging. However, label-free PAI signals from light absorbing chromophores within the tissue are nonspecific. The use of exogeneous contrast agents (probes) not only enhances the imaging contrast (and imaging depth) but also increases the specificity of PAI by binding only to targeted molecules and often providing signals distinct from the background.

**Aim:**

We aim to review the current development and future progression of photoacoustic molecular probes/contrast agents.

**Approach:**

First, PAI and the need for using contrast agents are briefly introduced. Then, the recent development of contrast agents in terms of materials used to construct them is discussed. Then, various probes are discussed based on targeting mechanisms, *in vivo* molecular imaging applications, multimodal uses, and use in theranostic applications.

**Results:**

Material combinations are being used to develop highly specific contrast agents. In addition to passive accumulation, probes utilizing activation mechanisms show promise for greater controllability. Several probes also enable concurrent multimodal use with fluorescence, ultrasound, Raman, magnetic resonance imaging, and computed tomography. Finally, targeted probes are also shown to aid localized and molecularly specific photo-induced therapy.

**Conclusions:**

The development of contrast agents provides a promising prospect for increased contrast, higher imaging depth, and molecularly specific information. Of note are agents that allow for controlled activation, explore other optical windows, and enable multimodal use to overcome some of the shortcomings of label-free PAI.

## 1 Introduction

How does the human body look on the inside? Well, it depends on how you look at it. The most direct way to look inside is to cut it open, e.g., through surgery. An alternative is to use less invasive methods such as using an endoscope. The beauty of medical imaging is that we can see inside the human body in ways that are less invasive than surgery or endoscopy. Developing a noninvasive imaging modality with high spatial resolution, real-time imaging, and deep penetration depth, while yielding both structural (anatomical) and functional information at low cost, is of prime interest. Some of the well-known medical imaging modalities such as x-ray projection imaging, x-ray computed tomography (CT), magnetic resonance imaging (MRI), single photon emission computed tomography (SPECT), positron emission tomography (PET), and ultrasound (US) imaging, have been widely used for clinical and preclinical imaging for decades.^1^^,^^2^ They have their own advantages and disadvantages. MRI is expensive and time consuming.^3^ X-ray, CT, and PET/SPECT use ionizing radiation, limiting their applications in continuous monitoring.^4^ Although US imaging is cost effective and real time, it has poor soft tissue contrast and lacks functional imaging capability.^5^ Therefore, scientists are always looking for a new imaging modality.

Optical imaging is a strong contender. It uses nonionizing radiation and can provide various types of tissue contrast (absorption, scattering, polarization, etc.) and high-resolution structural and functional information.^6^^,^^7^ There are various types of light-based imaging techniques, such as optical/fluorescence/two-photon/multiphoton microscopy, bioluminescence imaging, optical projection tomography, optical coherence tomography (OCT), diffuse optical tomography (DOT), and Raman imaging.^8^ However, strong absorption and scattering of light in biological tissue limits high-resolution imaging depth to ∼0.1  mm (∼mean-free path) in conventional optical microscopy. Imaging depth is slightly higher with the use of two-photon or multiphoton microscopes.^9^ OCT on the other hand can image up to the transport mean-free path (∼1  mm in biological tissue) as it uses ballistic and quasi ballistic photons for imaging. Eye imaging (retinal imaging) is one of the main application areas of OCT due to nearly absorption/scattering-free light traveling through the aqueous humor before it reaches the retina.^10^ OCT has also been used for skin imaging and other applications with up to 2-mm imaging depths.^11^ When light focusing is not essential, even the scattered photons can contribute to the imaging signal, resulting in higher imaging depth, with the sacrifice of spatial resolution. For example, using diffused photons in DOT, one can image up to several centimeters inside the body/brain (even through the skull); however, it has poor spatial resolution (∼0.5 to 1 cm).^12^ Due to strong absorption of light by biological tissue, beyond 5 to 7 cm, the scattered photons are so sparse that even the most sensitive detectors struggle to extract any useful signal for imaging. Hence, beyond this hard depth limit (∼5 to 7 cm in biological tissues), optical imaging becomes very challenging.

So, on the one hand, we have shallow imaging depth with high-resolution optical imaging (limited by the diffraction limit), and on the other hand, we have deep tissue imaging (∼5 to 7 cm) with poor spatial resolution. Photoacoustic imaging (PAI) bridges this gap by enabling deep tissue imaging yet maintaining relatively higher spatial resolution (resolution is scalable depending on the imaging depth).^9^^,^^13^^–^^19^ PAI achieves this by taking a hybrid approach of combining optical illumination with acoustic detection. As sound scattering in tissue is roughly two orders of magnitude less than light scattering,^9^ PAI can maintain high spatial resolution even at deeper imaging depths beyond the transport mean free path. PAI is based on the PA effect in which illuminating light energy is absorbed by tissue chromophores (such as melanin, collagen, lipids, hemoglobin, myoglobin, bilirubin, cytochromes, water, DNA, and RNA^20^^–^^25^) and under certain conditions produces local temperature rise (on the order of millidegrees), which in turn produces pressure waves. These pressure waves travel within the tissue and come out at the surface, where they are acquired using acoustic detectors. These acoustic waves (also known as PA waves) are later converted into an absorption map of the tissue with the help of various reconstruction algorithms.^26^^–^^35^ Aided with modern deep learning methods, the recovery of absorption maps has become more accurate and faster with better resolution and fewer artifacts.^36^^–^^44^ These absorption maps provide the structural/functional information of the tissue inside the body. Depending on how PAI imaging systems are engineered, they are commonly divided into a few subcategories, namely, PA nanoscopy,^45^^,^^46^ photoacoustic microscopy ,^47^^–^^52^ optoacoustic (PA) mesoscopy,^17^^,^^53^^,^^54^ photoacoustic endoscopy,^55^^–^^60^ preclinical photoacoustic computed tomography/photoacoustic tomography (PACT/PAT),^61^^–^^65^ and clinical PACT/PAT.^66^^–^^73^

Over the last two decades, PAI has been used for various preclinical and clinical applications.^14^ These include imaging of the whole body (small animal),^64^ brain,^61^^,^^66^^,^^74^ breast,^73^, vasculature,^69^^,^^75^^–^^79^ skin,^80^^,^^81^ eye,^75^ diabetic feet,^76^ zebrafish,^48^ chicken embryo,^82^ etc. In addition, real-time temperature monitoring^83^ and vulnerable plaque detection have also been demonstrated.^84^ Development of PAI was further enhanced with the use of optical probes or optical molecular probes specifically made for PAI. We call these probes PA probes or PA contrast agents.^85^^–^^91^ These exogeneous contrast agents/probes not only enhance the PA signal (in terms of signal strength) but also provide information about certain molecules (using targeting) that otherwise would not be visible under normal label-free PAI.^92^ The maximum imaging depth demonstrated so far without using any contrast agent (label-free) is 5 cm *in vivo*,^73^ whereas with the use of contrast agent, the imaging depth achieved so far is 12 cm *in vitro.*^93^^,^^94^ PAI can be combined with other imaging modalities (including other optical imaging modalities, such as fluorescence imaging, OCT, and two-photon microscopy) for multimodal imaging.^95^^–^^100^ PAI is also combined with other therapy tools such as photothermal therapy (PTT) or photodynamic therapy (PDT) for diagnosis and monitoring/treatment of disease. Hence, multimodal probes have also been developed to aid such imaging/therapeutic systems.^101^^–^^108^

In this review, we focus our attention on the development of these optical probes for deep tissue PAI and PA molecular imaging. The review is organized as follows: Sec. 2 describes the motivation for using an optical probe for PAI; Sec. 3 discusses PAI with different types of exogeneous probe based on their material composition; Sec. 4 presents activatable, photoswitching, and temperature-dependent PA probes; Sec. 5 discusses examples of molecular PAI; Secs. 6 and 7 describe contrast agents for multimodal imaging and PAI-guided therapy applications, respectively; Sec. 8 summarizes the review; and Sec. 9 provides our conclusions.

## 2 Motivation for Using Photoacoustic Probes

**Fig. 1:**
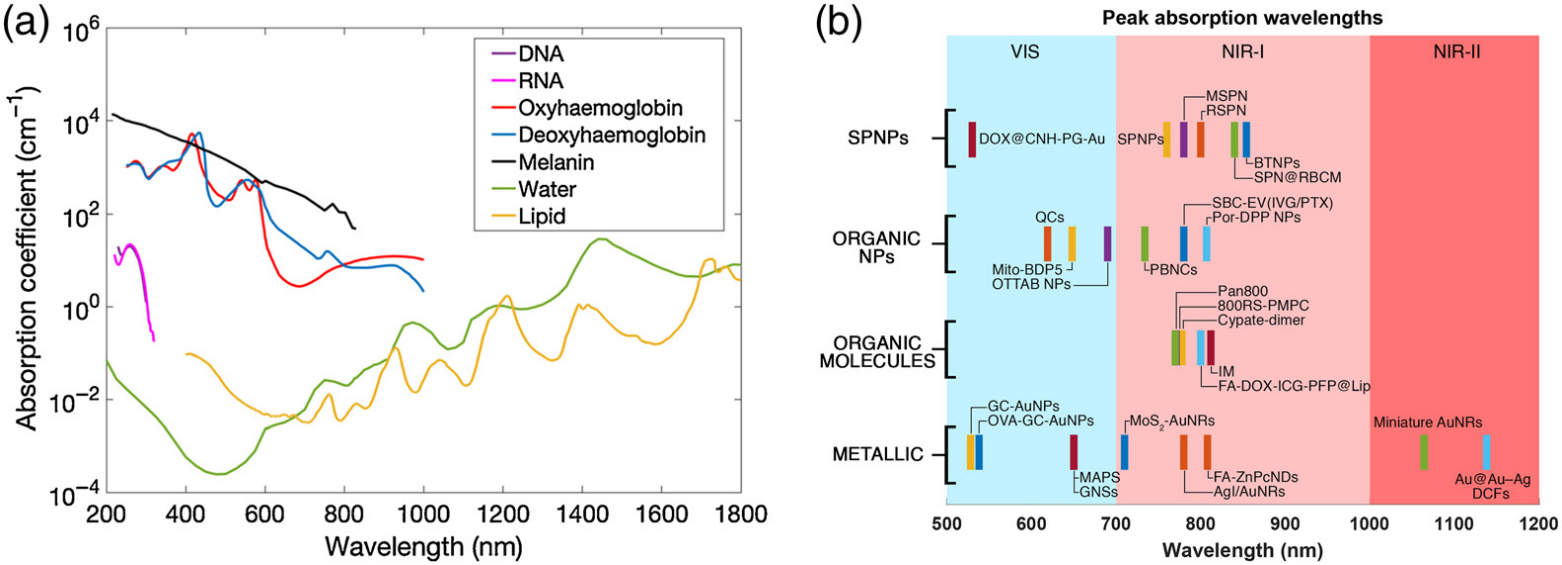
(a) The absorption spectra of various endogenous chromophores over the UV, visible, NIR-I, and NIR-II spectrum. Reprinted with permission from Ref. 109. (b) The peak absorption wavelengths of a select few molecular imaging probes categorized by their material composition. NPs, nanoparticles; SPNPs, semiconducting polymer nanoparticles.

Like other imaging modalities, the evolution of PAI is driven by a need for higher sensitivity and increased specificity. Accordingly, one of the first improvements is taking advantage of the various chromophores present in the body. These are shown in Fig. 1(a) with their optical absorption spectra in the UV, visible, and near-infrared (NIR) I and II regions. As we can see, oxy- and deoxyhemoglobin (present in the blood) strongly absorb light in the visible region. Utilizing this, green light (∼532  nm, a widely available laser wavelength with high pulse energy) was used extensively for vasculature imaging and oxygen saturation mapping (using multiple-wavelength PAI).^110^ The ultraviolet region (200 to 400 nm) also offers some intrinsic contrast such as cell nuclei (DNA, RNA absorb strongly in the UV region), allowing for label-free *in vivo* PAI of cell nuclei.^111^ Although UV and visible ranges (200 to 700 nm) produce strong intrinsic contrast, the imaging depth is limited due to the strong light scattering at shorter wavelengths and increased background absorption.^112^ Hence, PAI in the NIR region from 700 to 2500 nm offers a better proposition for deep tissue imaging as the light attenuation is low in the NIR range.^113^

The NIR window can be further split into the first NIR window (700 to 1000 nm, NIR-I) and the second NIR window (1000 to 1700 nm, NIR-II). The NIR-I window has been very popular for deep-tissue PAI due to the availability of high energy pulse lasers.^114^^,^^115^ In the past few years, the NIR-II window has been explored for various optical imaging applications.^116^^–^^123^ Using PAI at the 1064 nm wavelength, whole body mouse,^64^ rat,^64^ and human organ^69^^,^^73^ imaging has been demonstrated. Although the NIR window provides less attenuation of light, it also poses another challenge for PAI: the imaging contrast suffers. Therefore, PAI in the NIR window (both NIR-I and NIR-II range) using intrinsic absorbers is challenging. Although blood absorption is less in the NIR window compared with the visible window, the NIR window is still used for blood vessel imaging due to the deeper imaging depth in the NIR window. On the other hand, melanin absorbs light strongly in the NIR-I window; hence melanoma imaging in the NIR-I window is very effective with high contrast. Nevertheless, PAI with endogenous contrast is often not strong enough, especially in the NIR region. It also does not allow for highly specific imaging of molecules that may be of greater interest.

Such factors have led to the development of optical agents that help increase sensitivity and contrast. Over the last two decades, researchers have developed various types of probes to interrogate the body. These are primarily based on metallic [e.g., gold nanoparticles (AuNPs)], inorganic [e.g., carbon nanotubes (CNTs), quantum dots], organic small molecules [e.g., indocyanine green-ICG, IRDye800, methylene blue (MB)], semiconducting nanoparticles (NPs), reporter genes (RGs), and others ^91^^,^^108^^,^^124^^–^^139^ These probes have helped facilitate deeper imaging across various forms and applications of PAI.^140^ Similar to endogenous contrast, some probes may perform best only in specific absorption windows. Although, some of these probes have broad absorption in the NIR-II window, PAI is typically done with a 1064 nm wavelength in most cases.^118^ This is due to the availability of 1064-nm pulsed lasers (e.g., Nd:YAG laser) in the market. There are also some advantages of the NIR-II window for PAI. Regarding safety, the NIR-II window has higher light exposure limits (higher maximum permissible exposure^141^); therefore, more light energy can be used for imaging and better imaging depth can be achieved.^142^ Longer wavelengths also induce less tissue scattering and enhanced tissue penetration. Incidentally, oxyhemoglobin absorption is high, at around 1064 nm, leading to deeper levels of tissue oxygenation imaging.^143^ There are also challenges, especially from water absorption (the human body has a high water composition), which is quite strong around 1400 to 1500 nm.^121^ Therefore, not all wavelengths in NIR-II may be useful for PAI. So far, mostly shorter wavelengths in the NIR-II window (1000 to 1400 nm) have been demonstrated for PAI.^51^^,^^144^^,^^145^

Naturally, the next progression tackles specificity in the form of agents that can target specific molecules. Because base PAI utilizes optical absorption of tissues for contrast, it cannot identify abnormal from normal cells unless the cells are overexpressing chromomeric markers (e.g., melanoma^146^) or are labeled by targeted contrast agents to enhance the contrast between normal and targeted pathological tissues.^147^ As such, molecular contrasts agents that are able to identify specific biomarkers can in turn provide more specific PA signals from the anomalous material. This specific PA signal can be easily filtered out from background signals or areas where the biomarker is not present. In addition, the excitation ranges of these probes can be tailored to further distinguish them from known endogenous contrasts or even nontargeted agents in the same vicinity.

## 3 Photoacoustic Imaging with Exogeneous Probes

The NIR window has been extensively employed for deep tissue imaging at the sacrifice of imaging contrast as the absorption of intrinsic chromophores in the NIR region is low. Therefore, contrast agents are in great demand for NIR PAI. So far, metallic NPs, small organic molecules, organic NPs, semiconducting polymer (SP) NPs, carbon materials, fluorescent proteins, RGs, etc., have been demonstrated as PA contrast agents.^86^^,^^91^^,^^118^ These can be used either by targeting a specific receptor/protein or can be used as nontargeted passive imaging agents. Nontargeted imaging agents may extravasate from the blood stream due to increased vascular permeability or the enhanced permeability and retention (EPR) effect in tumor tissue, whereas targeted imaging agents target a specific molecule, such as receptors, proteins, or enzymes.^148^ Although all contrast agents have their own absorption wavelengths and abundant choices of materials for PAT contrast agents,^149^ not all choices can be considered useful for PA probes. Photostability, renal clearance, optical absorption, and photothermal conversion efficiency are four vital parameters for assessing effective contrast agents, and therefore, they should be taken into consideration when designing new materials.

Commonly used contrast agents can be classified into (a) inorganic agents, such as metallic NPs or carbon-based nanomaterials, and (b) organic agents, such as organic dyes and SP NPs. A few of the probes discussed later in this review are sorted by material and their peak absorption wavelengths and shown in Fig. 1(b). Inorganic agents show high photothermal conversion efficiency and photostability, whereas organic agents show good biocompatibility and biodegradability. In this section, metallic NPs are discussed first, followed by organic dyes and organic NPs. Semiconducting polymer NPs, and carbon-based nanomaterials are presented at the end of this section.

### 3.1 Metallic Nanoparticles

As one class of strong optical-absorption nanomaterials, metallic NPs have been widely applied as exogenous contrast agents for PAI. The strong tunable absorption of metallic NPs is primarily due to the localized surface plasmon resonance (LSPR) effect. The LSPR effect occurs when conduction electrons oscillate in concert with the electromagnetic field, leading to optical absorption that is five orders of magnitude greater than dyes.^89^^,^^150^^,^^151^ The shape and size of particles are two factors that can influence the resonance frequency of the oscillation. For example, longer nanorods have peak absorption at longer wavelengths.^89^

AuNPs are one major category of metallic NPs. The unique physicochemical properties, stronger light absorbance, stronger scattering than other materials, and better biocompatibility of AuNPs have promoted their use in molecular imaging. AuNPs allow for noninvasive remote sensing of the characteristics of biological processes and interactions at the molecular level. It has great potential for early disease detection as aberrations occur at the cellular and molecular levels well before anatomical changes.^152^ It was reported that AuNPs modified with two conjugated molecules [c(RGDyk)-MHDA and LSC] can be employed for tumor-targeting imaging.^153^ The smart NPs were stabilized at the pH of normal tissues but aggregated in tumor tissues for achieving selective targeting and imaging. Enhanced contrast was shown for both *in vivo* and *ex vivo* PAI. Another AuNP-based probe (Au-RRVR) was also presented for tumor imaging activated by both furin enzyme and acidic pH.^154^ It not only showed enhanced accumulation in tumor microenvironment via PAI but also enhanced the PTT effect for therapy of tumors.

Commonly used AuNP structures include gold nanospheres (AuNSs),^155^^–^^157^ nanorods,^152^^,^^158^^–^^160^ nanoshells,^161^ nanoprisms,^162^ nanocages,^163^ nanobeacons,^164^^,^^165^ nanostars,^166^^,^^167^ and nanovesicles.^168^ An earlier work presented that AuNSs could be utilized to detect and differentiate cancer cells at multiple wavelengths.^157^ In addition, when coated with glycol-chitosanm, AuNS can help cancer cell imaging with enhanced PA signals.^156^ However, AuNSs with a diameter of around 20 nm show an absorption peak at ∼522  nm, which limits their utility for deep-tissue PAI.^169^ Gold nanoshells, which are composed of a spherical silica core and gold shell, have also been employed as PA contrast agents. A PA molecular probe based on the conjugation of gold nanoshells and VCAM-1 antibody was proposed for atherosclerotic plaque imaging.^161^ However, nanoshells showed more scattering than absorption and a broad optical spectrum, making them less effective than gold nanorods (AuNRs).

AuNRs show characteristic surface plasmon resonance along the longitudinal direction leading to one absorption peak in the NIR-I region (∼698  nm). The longitudinal mode redshifts as the aspect ratio increases.^169^ Due to its strong NIR absorption, high photothermal conversion efficiency, and inertness, AuNRs were widely applied in PAI with the surface modified by other materials. Due to thermodynamic instability, bare AuNRs in their original state often undergo shape shifts under laser irradiation, resulting in a decrease in PA signal after a few seconds of imaging. Therefore, modification of AuNRs is sometimes needed to obtain a more stable and consistent PA signal. An interesting nanohybrid, AuNR-melanin nanohybrids with tunable polydopamine (PDA) coating, was demonstrated for PAI in the second NIR window.^170^ The PDA-enabled strategy resulted in PA signal enhancement and stability, but at the cost of large-size nanohybrids. The AuNRs coated with PDA (diameter>40  nm) had a much larger size than pristine AuNRs (∼12.46  nm diameter).

**Fig. 2:**
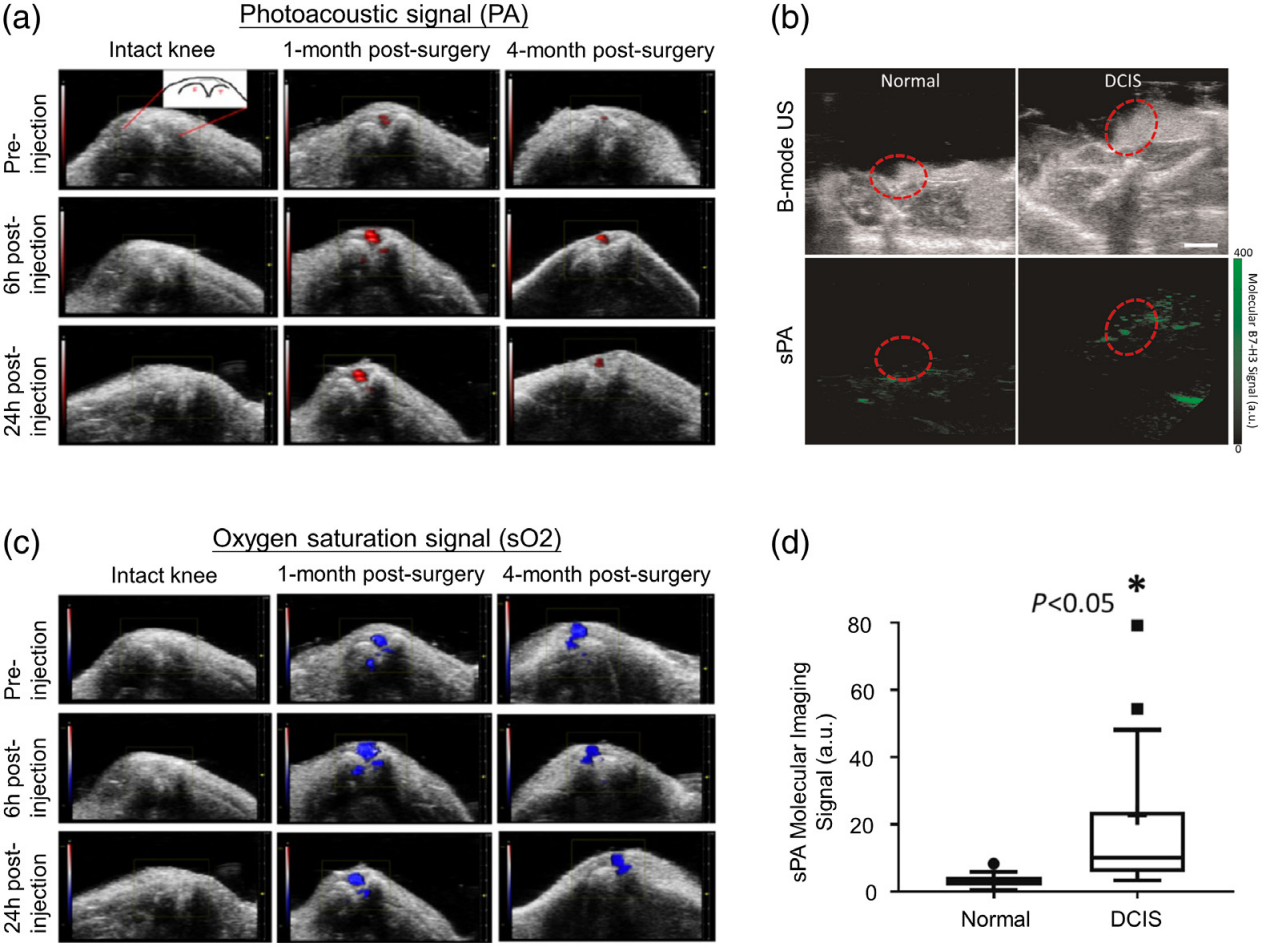
(a) US-PA overlay images of intact knees from unoperated mice and surgically operated knees from surgical DMM-induced posttraumatic OA mice at different time points. The PA images of surgically operated knees were recorded 1 month and 4 months after surgery. (b) Under laser illumination of 750 and 850 nm, US-PA images of oxygen saturation in synovial blood before and after injection in intact knee and postsurgical OA knees. (a), (b) Reprinted with permission from Ref. 171. Copyright 2021 ACS. (c) After injection of B7-H3 Ab-ICG, B-mode US images (top) and spectroscopic PA images (bottom) of murine mammary glands with normal (left) or DCIS (right) tissues (red dashed circle). Scale bar=2  mm. (d) Box plot of spectroscopic PA signal (a.u.) distribution in normal mammary glands (n=17, spectroscopic PA=3.2±2.0 a.u.) and glands containing DCIS (n=53, spectroscopic PA=22.7±40.2 a.u.). Box plot follows Tukey rules. (c), (d) Reprinted with permission from Ref. 172.

Another interesting nanoprobe was designed for PA pain imaging and NIR imaging-guided PTT.^171^ Osteoarthritis (OA) is one of the most common reasons for painful conditions in the elderly, and precise localization and imaging of pain generators is in great demand. In this work, an anti-NGF-${\mathrm{MoS}}_{2}$-AuNPs nanocomplex was synthesized via electrostatic interaction between AuNRs and small ${\mathrm{MoS}}_{2}$ and then covalent interaction between anti-NGF antibody and ${\mathrm{MoS}}_{2}$-AuNRs. After interaction with a negatively charged ${\mathrm{MoS}}_{2}$, ${\mathrm{MoS}}_{2}$–AuNRs showed great photothermal stability and higher PA signals under different wavelengths than bare AuNRs. It also showed concentration-dependent PA signal generation under 710-nm laser irradiation. For actively targeted-pain imaging, ${\mathrm{MoS}}_{2}$–AuNRs were conjugated with anti-NGF antibody as nerve growth factor (NGF) plays a vital role in peripheral nerve activation and sensitization. To evaluate the effectiveness of the nanocomplex, noninvasive pain imaging was done *in vivo*. A well-received post-traumatic OA mouse model, induced by surgical destabilization of medial meniscus (DMM), was injected with anti-NGF-${\mathrm{MoS}}_{2}$-AuNP intravenously. Under 710-nm laser excitation, the nanoprobes accumulated in OA knee joint 1 and 4 months after surgery but presented no PA signal in intact knees, indicating its effective targeting ability at the inflamed regions with abundant NGF [Fig. 2(a)]. Oxygen saturation (${\mathrm{sO}}_{2}$) analysis was also performed to differentiate the PA signal produced by the anti-NGF-${\mathrm{MoS}}_{2}$-AuNP and by the hemoglobin, which is one type of endogenous chromophore that produces background noise. The outcomes suggested that ${\mathrm{sO}}_{2}$ remained almost unchanged in the OA knee before and after nanocomplex injection, as shown in Fig. 2(b). This demonstrated that the variations of PA signals at 710 nm were due to the accumulation of the NPs. In addition, under imaging guidance, PTT could alleviate pain and improve motor function in a well-received posttraumatic OA mouse model. These results exhibit the potential of anti-NGF-${\mathrm{MoS}}_{2}$-AuNP for *in vivo* pain imaging and targeted photothermal treatment. In addition, AuNRs also found interesting applications in sensing chemical species^158^ and mapping matrix metalloproteinase-2 (${\mathrm{MMP}}_{2}$) in atherosclerotic plaques.^173^

Despite promising a potential and sharp increase in various preclinical uses of AuNPs, few contrast agents based on AuNPs have received Food and Drug Administration (FDA) regulatory approval compared with other small molecules. Thus, the translation of the AuNPs from bench to clinics has been limited. The size and physical properties are usually used to explain the limitations of clinical translation; these are related to renal clearance and associated inflammatory effects.^174^ The cores of many proposed AuNPs are >20  nm, higher than the renal clearance threshold (5 to 10 nm), and the retention of nonbiodegradable AuNPs can result in unfavorable behavior *in vivo*. The safety concern could be addressed by the development of miniaturized AuNPs. Previously, ultrasmall AuNPs were proposed as molecularly activated plasmonic nanosensors, which enhanced the PA signals in cancer cell imaging.^155^ Miniature hollow AuNRs (∼46  nm in length) were also reported to show higher PA signal intensity [∼3.5-fold higher than the larger (∼105  nm in length) hollow AuNRs] in the NIR-II window.^175^ Another miniature AuNRs-based probe was presented as an NIR-II contrast agent for tumor imaging.^159^ Seedless approaches were employed to reduce the size from dimensions of 18 nm by 120 nm to dimensions of 8 nm by 49 nm. This improved the photothermal stability and produced strong PA signals in the NIR-II window with a 30% improvement in efficiency of nanoparticle delivery to tumors in living tumor-bearing mice.

In addition to AuNPs, other metallic NPs have also been used as PA probes, e.g., silver-, copper-, Fe-, and nickle-based NPs. In the past, silver nanoplates were used for *in vivo* cancer imaging.^176^ However, the toxicity of silver NPs hinders their extensive use as a contrast agent. Fe-based nanoprobes were also prepared for tumor imaging.^177^ $\mathrm{Fe}@{\mathrm{Fe}}_{3}O_{4}$ NPs were synthesized and then conjugated with a c-RGD peptide to achieve active tumor targeting ability. Due to their tunable physical, chemical, and optical properties, copper-based nanomaterials have attracted greater attention. Gold-based NPs often show characteristic absorption peak in the NIR-I region, whereas copper-based NPs usually exhibit a strong absorption peak over 1000 nm, which belongs to NIR-II region.^178^ By utilizing NIR-II laser excitation, a higher penetration depth and lower background noise can be achieved, making copper-based NPs promising for PA deep tissue imaging. For example, copper sulfide nanoparticles (CuS NPs) with an absorption peak at 990 nm allowed for brain visualization and lymph node (LN) imaging with an Nd:YAG laser at 1064 nm.^138^ Polyethylene glycol (PEG)-coated CuS NPs were also reported as a contrast agent of PAI with their peak absorption tuned to 1064 nm.^179^ By adjusting the sulfide/copper from 3:1 to 0.75:1, the peak absorption of Cus NPs was tuned from ∼1000 to ∼1300  nm.^180^ After doping magnetic ${\mathrm{Ni}}^{2+}$ ions into CuS NPs, dual-modal NIR-II PA/MRI was achieved. The NIR-II window was also explored by Park et al.,^181^ who designed nickel dithiolene-based polymeric nanoparticles (NiPNP) that showed strong absorption at 1064 nm, good biocompatibility, and deep penetration capability. *In vitro* experiments were performed to show the PA characteristics of NiPNP, further confirming that the PA spectrum was nearly the same as the optical absorption spectrum. NiPNP also showed linear concentration-dependent PA signals. Via the dual-US/PA imaging system, the overlaid images demonstrated that the maximum imaging depth was around 5.1 cm. *In vivo* PAI of SLN, gastrointestinal (GI) tracts and bladders of live rats were also presented. These images, acquired after injecting the NiPNP, indicated that the SLN and GI tracts were clearly visualized with an enhanced signal and ∼1.5  cm imaging depth, whereas the bladders were visible even at 3.4 cm in depth.

Another instance of nickel-based probes is the development of metallic microrobots used to image and navigate vasculature.^182^ These 5- to 20-μm-diameter cell-sized microrobots consist of a silica core with nickel, gold, and Lipo-ICG coatings. To facilitate safe travel in capillaries, the diameter of microrobots should ideally be around 5 to 10  μm. These microrobots exhibited peak absorbance around 780 nm with signals from particles above 10  μm easily distinguishable from blood. *Ex vivo* tests in a vascular network exhibited the probes’ exceptional ability of real-time PAI guidance as well as magnetic manipulation in flowing blood. *In situ* tests on murine cerebral vasculature demonstrated three-dimensional (3D) volumetric imaging as well as the same magnetic manipulation in a dynamic environment. Although manipulation was not strong enough for upstream travel, the probes consistently showed high contrast with their surrounding environment for real-time imaging. In addition, the Lipo-ICG coating could allow future versions to transport drugs to specific downstream locations.

Broadly, metallic NPs show high photostability, high conversion efficiency from absorbed light to heat, and high molar absorptivity. On the flip side, biocompatibility and biosafety issues need to be addressed to translate them into the clinic. This has also stimulated the interest in repurposing FDA-approved organic dyes for PAI, which is discussed below.

### 3.2 Small Organic Molecules

For small organic molecules to be used as PA contrast agents, they should have absorption in the NIR window, allowing for maximum depth of light penetration in tissues. Many of these organic dyes also have fluorescence and can be used in fluorescence imaging applications as well. Under laser irradiation, the absorption of light energy makes the electrons in the dye molecule jump to excited states. When they come back to the ground state, the dye molecules at the singlet excited state could dissipate the energy in three different pathways. The direct radiative decay from the singlet excited state to the ground state generates fluorescence signal. Otherwise, molecules could transit to the lowest triplet state and then relax to the ground state through radiative decay, known as phosphorescence. By contrary, there could also be a nonradiative vibration relaxation to the ground state, resulting in heat generation. PA signal generation is mainly based on this process of converting light energy to heat for local temperature rises and acoustic wave generation.^183^ The fraction of the absorbed energy that is reradiated as fluorescence is called fluorescence quantum yield (QY). Molecules with high fluorescence QY are good for fluorescence application, whereas NIR dyes with low fluorescence QY can be good candidates for PA contrast agents. Commonly used fluorescence dyes include ICG,^172^^,^^184^^,^^185^ MB,^186^ Evans blue,^187^ IRDye800CW,^24^^,^^188^ Alexa Fluor750,^189^^,^^190^ MMPSense 680,^191^ NIR caspase-9 probe,^147^ 800RS^192^ etc. Except for ICG, Evans blue, and MB, the other the contrast agents are not FDA-approved for human use; hence, they can be evaluated only in phantoms or animal models.

ICG can serve as a contrast agent in both PAI and fluorescence imaging. It has a concentration-dependent absorption band from 700 to 900 nm and low fluorescence QY, leading it to be an effective PA contrast agent. However, due to its fast clearance from the body,^193^ it needs to be functionalized to enable molecular targeting and longer circulation time in the body. For example, an anti-B7-H3 antibody conjugated with ICG (B7-H3-ICG) can identify normal breast tissue from breast cancer.^172^^,^^184^^,^^185^ Ductal carcinoma *in situ* (DCIS) is a preinvasive lesion, which may lead to invasive carcinoma; thus most patients with DCIS undergo treatment such as breast-conserving surgery. But a large proportion of low-grade DCIS and some high-grade DCIS will not progress to cancer, and overtreatment is possible for a portion of patients. Molecular imaging of DCIS can potentially reduce unnecessary treatment. B7-H3 was found to be overexpressed in the four subtypes of breast cancer, but normally expressed in benign tissues. Previously, B7-H3-ICG was designed to specifically target B7-H3 and monitor its expression level in DCIS to differentiate low- and high-grade lesions.^172^ PAI was conducted in a transgenic mouse model, showing higher average PA signals for confirmed DCIS than for normal tissue [Fig. 2(c)]. The signal distribution in different tissues was shown in Fig. 2(d), indicating statistically significant higher PA signals for DCIS. Similarly, fluorescence imaging presented higher fluorescence signals for carcinoma regions. These results suggested effective differentiation between DCIS and normal mammary glands. Apart from being conjugated with anti-B7-H3 antibody, ICG can also be conjugated with antiepidermal growth factor receptor (EGFR) monoclonal antibody (Pan) for targeting cancer-associated EGFR.^194^ The resulting probe (Pan-EG4-ICG) showed high EGFR-specific PA signals in EGFR-positive cells *in vitro* and increasing PA signal in A431 tumors *in vivo.* MB is another FDA-approved contrast agent used for PAI that possesses low toxicity and wide absorption in the NIR-I window with a peak at 660 nm.^195^ Employing MB during PAI was shown to be effective for *in vivo* sentinel lymph node (SLN) mapping.^186^^,^^196^^,^^197^ The PA image after MB injection showed clearly visible SLNs compared with the image acquired before injection.

**Fig. 3:**
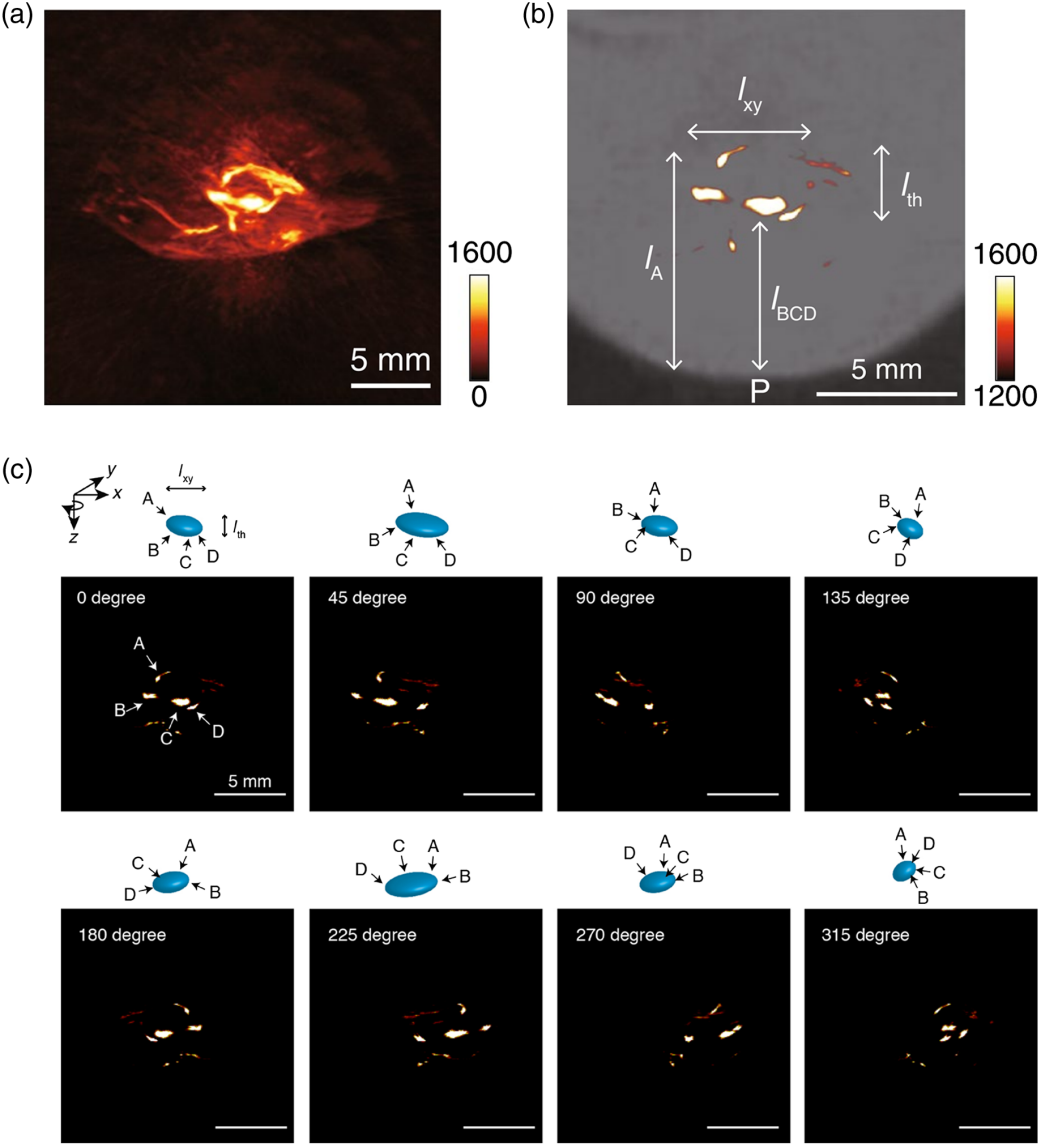
PA image of a tumor-bearing mouse injected with 800RS-PMPC (2.0  μmol/kg=40  nmol/20-g mouse) (a) under high-sensitivity conditions with a sensitivity scale from 0 to 1600 or (b) under low-sensitivity conditions with a sensitivity scale from 1200 to 1600. The PA image in (b) was overlaid with a bright-field image. (c) The projection on the xz plane of the 3D PA image of a tumor-bearing mouse injected with 800RS-PMPC. The images were shown at different angles with respect to rotation around laser path (z). Scale bar: 5 mm. Reprinted with permission from Ref. 192.

As a derivative of ICG, IRDye800CW is also an effective PA contrast agent, and it can be used for detecting LN metastases when conjugated with an antibody,^188^ monitoring glucose metabolism when conjugated with 2-deoxyglucose (2DG)^24^ or imaging tumor-related receptors when conjugated with a peptide.^198^ Anti-EGFR antibody conjugated with IRDye800CW (pan800) can be employed as a fluorescence/PA dual-modal contrast agent for the identification of metastatic LNs.^188^ After injecting pan800 1 to 5 days before surgery, metastatic LNs were identified from normal LNs in *ex vivo* nodal specimens obtained from head and neck squamous cell carcinoma patients via dual-modal imaging. In addition, Alexa750, MMPSense 680, NIR caspase-9 probe, and 800RS were also employed as PA probes. For example, a matrix metalloproteinases (MMP)-activatable PA probe composed of a quencher (BHQ3), polyarginine, Alexa750, and the polyglutamic acid chain was proposed for detecting follicular thyroid carcinoma.^189^ Alexa750-labeled Herceptin antibody was designed for assessing HER2 expression in breast cancer.^190^ In addition, as increased expression of MMPs is related to atherosclerotic plaque instability. MMPSense680 as an MMP-sensitive activatable fluorescent probe was also used for mapping MMP activity in the vulnerable plaque.^191^ An NIR fluorescent probe, which conjugated a fluorochrome (IR780) to Z-Val-Ala-Glu, was synthesized to monitor apoptosis caused by anticancer drug treatment.^147^ Another NIR cyanine dye, 800RS, was also used for PAI. Earlier work showed that using 800RS-PMPC, coupled 800RS with PMPC, 3D visualization of tumor can be achieved in mice.^192^ The probe effectively and selectively accumulated in the tumor and generated strong PA signals that were distinguished from background noise signal produced by endogenous hemoglobin [Fig. 3(a)]. The PA image of tumor tissue pretreated with 800RS-PMPC is shown in Fig. 3(b), with suppressed hemoglobin, low sensitivity, and high threshold. Figure 3(b) shows the projection of the 3D image on the xz plane; the vertical distance from point P means depth. The shallow three points [B, C, and D in the top-left panel of Fig. 3(c)] were at a depth of $l_{\mathrm{BCD}}=∼5\;\mathrm{mm}$ from point P, whereas point A was deeper ($l_{A}=∼8\;\mathrm{mm}$) and less bright. The four points (A, B, C, and D) were accommodated within a volume, which was located ∼6  mm beneath the skin and with a thickness of $l_{\mathrm{th}}=∼3\;\mathrm{mm}$ and a horizontal/longitudinal (xy) width of $l_{xy}=∼4\;\mathrm{mm}$. the z (laser path) axis was rotated at angles of 0 deg, 45 deg, 90 deg, 135 deg, 180 deg, 225 deg, 270 deg, and 315 deg; the PA signal distribution in the xy plane is shown in Fig. 3(c). The thickness ($l_{\mathrm{th}}$) of the volume, which included the four points, remained unchanged at ∼3  mm during the rotation, whereas the xy width ($l_{xy}$) changed as the tilt angle changed, with the shortest distance (3 mm) at 135 deg and 315 deg and the longest (6 mm) at 45 deg and 225 deg. The four bright spots shown in Fig. 3(c) were encompassed in the same volume, which exhibited an ellipsoidal shape similar to the injected tumor. The above-mentioned results suggest that the probe 800RS-PMPC possessed the ability to define both front and rear surfaces of the tumor and allowed for 3D imaging of the tumor.

Organic dyes have the merits of good biodegradability, biocompatibility, low toxicity, and relatively small size, making them an attractive PA probe for molecular imaging.^86^ But the low photostability, partial fluorescent output, and relatively low PA conversion efficiency compared with NPs has hindered the use of organic dyes. To address these problems, the dimerization of NIR cyanine molecules was reported as an effective method to create photostable molecules without increasing the dose.^199^ Cypate, which is an NIR fluorescent dye with an absorbance peak at 778 nm, was considered for the PA probe for this experiment. After carbon–carbon (C–C) dimerization, a small hypsochromic shift of the peak absorption with shoulder peak increase was observed, and more than 90% fluorescence quenching was achieved. To explore whether the conjugation of peptides with cypate would affect the dimerization, a tumor-avid cypate-peptide conjugate, LS301, was used. Under multiple wavelengths, cypate dimer and LS301 dimer produced a stronger PA signal than their corresponding monomer. In the same concentration condition, LS301 dimer also showed a higher PA amplitude and a higher rate of PA signal change versus concentration than LS301 and ICG. These outcomes suggested that the dimerization of NIR cyanine molecules could be a potential method for tackling the intrinsic problems of organic fluorescent dyes.

### 3.3 Organic Nanoparticles

Contrast agents are expected to possess high photostability, high conversion efficiency from absorbed light to heat, and high molar absorptivity. Metallic NPs are widely applied for PAI as they possess some of these characteristics, leading to strong PA signal generation. However, the need to translate PAI probes to the clinic stimulated the interest in repurposing FDA-approved organic dyes for PAI agents. However, the low photostability, partial fluorescent output, and relatively weaker PA signal generation compared with metallic NPs hindered widespread use of organic dyes. One common approach to overcoming these issues is to encapsulate organic dyes inside the NPs, providing size-independent photophysical properties and thus the opportunity to develop PAI probes for various excitation wavelengths. Such dye encapsulation methods have several advantages. First, the organic components encapsulated within biocompatible polymers to neutralize their surface can lead to ideal biodistribution for *in vivo* applications. Second, the NP surface can be functionalized for specific purposes, e.g., attaching a targeting antibody for cancer detection.^200^ Third, the image contrast can be improved due to the high concentration of dye in the nanoparticle.^200^ Finally, higher photostability can be achieved. In addition to dye encapsulation, other new organic NPs were also designed to perform high-quality PAI. Some of the applications of organic NPs include tumor imaging,^201^^–^^206^ MMP mapping,^207^ cancer cell targeting,^200^ mitochondrial imaging,^208^ and SLN detection.^209^

As one of the most frequently used organic dyes, ICG has been employed in PAI due to its high absorption in the NIR region. Its fast clearance, a relatively weak PA signal strength compared with inorganic NPs, and partial fluorescent conversion are some of the challenges. Dye encapsulation holds great prospects for tackling these problems. For example, an agent was proposed by encapsulating ICG into a biocompatible matrix. This ICG-embedded nanoparticle was conjugated with HER-2 antibody on its surface for cancer targeting, leading to specific binding to cancer cells as shown by *in vitro* PAI.^200^ In addition, ICG was encapsulated into extracellular vehicles (EVs) with paclitaxel and sodium bicarbonate, which was employed as a sonosensitizer to perform PAI-guided sonodynamic therapy.^201^ Good photostability and cellular uptake can be achieved with these NPs, which also showed effective accumulation in tumor and thus presented high-contrast, high-resolution PAI.

**Fig. 4:**
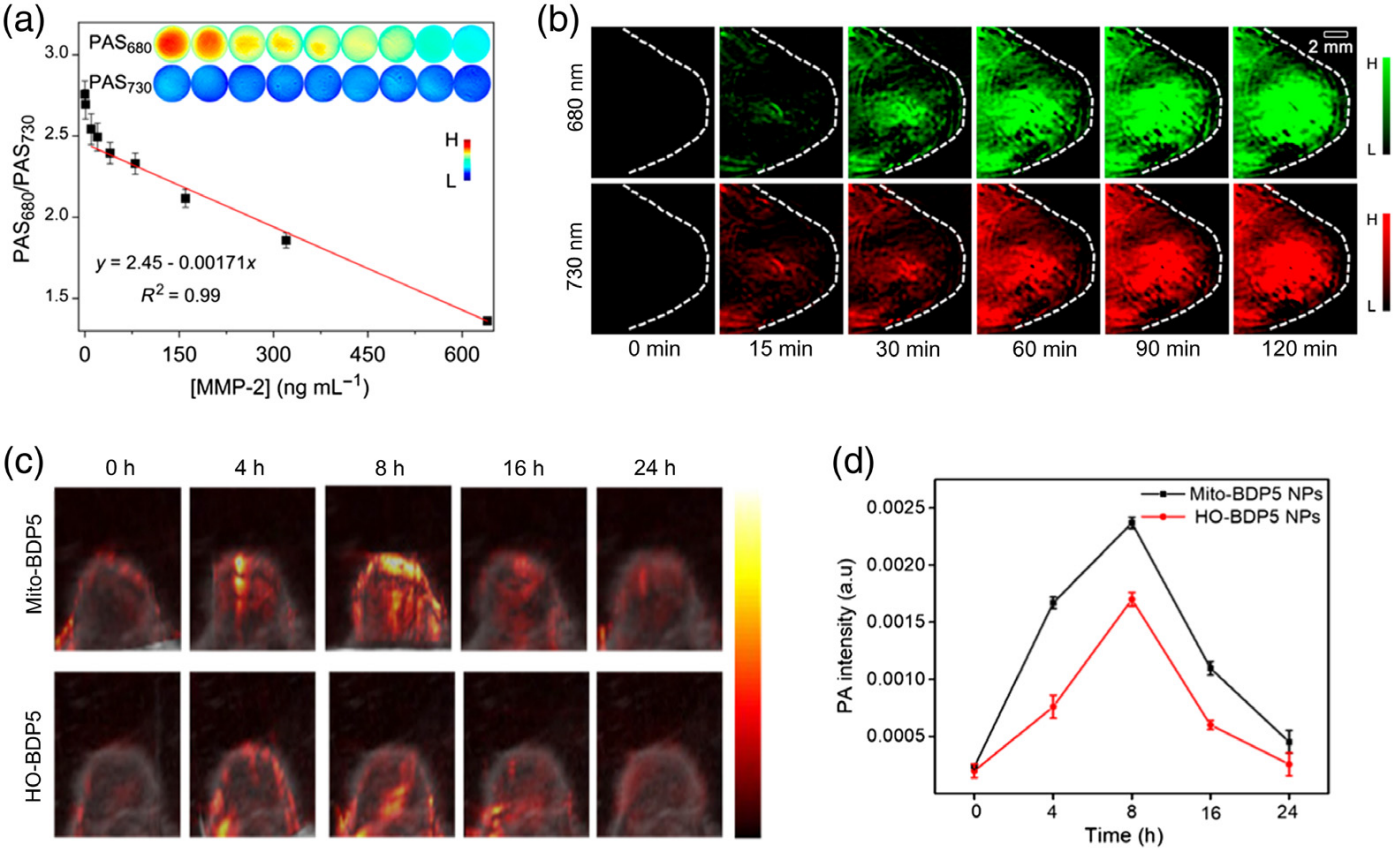
(a) The ratio of PA signal at 680 nm to that at 730 nm of the QC probe (0.25  μM) as a function of the MMP-2 concentration recorded at 37°C (inset: PA images of the QC probe solutions with different concentrations of MMP-2). (b) Under laser illumination of 680 and 730 nm, PA images of 4T1 tumor-bearing mice that were injected with the QC probe (60  μM, 200  μL). The tumor regions are marked by white dotted circles. (a), (b) Reprinted with permission from Ref. 207. Copyright 2019 American Chemical Society (ACS). (c) PA images of mouse tumor sites at different time points. The tumor-bearing mice were injected with HO-BDP5 NPs and Mito-BDP5 NPs in the tail vein. (d) PA signal intensity from the tumor sites at different time points. The tumor-bearing mice were injected with HO-BDP5 NPs and Mito-BDP5 NPs in the tail vein. (c), (d) Reprinted with permission from Ref. 208. Copyright 2021 ACS.

Other organic NPs have been developed without the encapsulation technique. An organic nanoparticle (QSY21-GPLGVRGY-Cy5.5, also denoted as QC), composed of an NIR dye Cy5.5, a quencher QSY21, and a peptide substrate of MMP-2, was designed for noninvasively detecting MMPs *in vivo*.^207^ The nanoparticle not only was selectively activated by MMP-2 due to MMP-2 cleavable peptide sequence but also performed well in dual-fluorescence imaging/PAI. Simultaneously, the nanoparticle was used as ratiometric probe because the cleavage of the peptide linker changed the aggregation state of the molecules, leading to a tunable absorption at around 680 nm but an unchanged absorption at around 730 nm. In the presence of MMP-2, the peptide sequence GPLGVRGY was cleavable, and Cy5.5 was separated from the quencher. The NPs produced linearly decreasing PA signals against MMP-2 concentration at 680 nm and nearly unchanged PA signal at 730 nm, which is shown in Fig. 4(a). Taking the PA signal at 730 nm as a reference, the ratiometric probe offered quantitative detection of MMP-2 expression. In addition, PA molecular imaging was performed on 4T1-tumor bearing mice, presenting significant and increasing PA signals in the tumor region [Fig. 4(b)]. Another interesting PAI organic nanoparticle was developed based on perylene tetracarboxylic diimide. These NPs showed effective accumulation in brain tumor and therefore enhanced PA signals at the orthotopic tumor sites.^205^ In addition, a porphyrin-based organic nanoparticle (Por-DPP NP) was synthesized for tumor imaging.^202^ Tumor-bearing mice were employed to assess the effect of Por-DPP NPs. After injecting Por-DPP NPs, the tumor site of mice showed a stronger PA signal than pre-injection. The agent not only performed well in tumor imaging but also could be used for PAI-guided PTT. Under 808-nm laser irradiation, the temperature of the tumor site that had been injected with Por-DPP NPs increased significantly, resulting in destruction of tumor cells and inhibition of tumor growth. The thick skull and scalp always pose a challenge in brain tumor imaging, especially with visible light leading to low penetration depth through the scalp and skull. Therefore, the NIR-II window was developed for precise PAI of brain tumor. Guo et al.^203^ presented a biocompatible and photostable NIR-II conjugated polymer nanoparticle that possessed the ability to perform accurate brain tumor imaging with deep penetration through the scalp and skull. In addition to PAI, the NPs were also used for PTT to inhibit the tumor progression.

Biocompatible NPs developed from nanoprecipitation of biliverdin were used as NIR PAI probe for SLN detection.^209^ *In vivo* PAI in mice showed the ability of these NPs to effectively accumulate in LNs, and mass spectroscopy was used to suggest that they can be completely biodegraded in the presence of biliverdin reductase. BODIPY, a fluorescent labeling material, has the advantages of a high molar extinction coefficient, good photostability, and narrow emission peak. Wang et al. developed a series of mitochondria-targeted BODIPY NPs with a cationic triphenylphosphine group (Mito-BDP1-5 NPs).^208^ Among them, Mito-BDP5 NP presented high photothermal conversion efficiency and high photo-induced cytotoxicity, resulting in being a good candidate for dual-modal agent (PTT and PAI). PAI was used to monitor the effectiveness of nanoparticle accumulation in the tumor site of tumor-bearing mice [Fig. 4I]. After injecting Mito-BDP5 NPs, the PA signal increased significantly and reached a maximum at 8 h, followed by a rapid decline to the initial level at 24 h [Fig. 4(d)]. This outcome suggested that the NPs not only effectively and rapidly accumulated in the tumor but also can be fully metabolized by the body.

### 3.4 Semiconducting Polymer Nanoparticles

Semiconducting polymer nanoparticles (SPNs) are advanced organic materials that have been applied in many imaging modalities, including fluorescent imaging, US imaging, and PAI. The advantages of SPNs are numerous and include large absorption coefficient, tunable absorption, high photostability, low toxicity,^86^ high structural flexibility, small size, and higher PA signal generation than single-wall carbon nanotubes (SWNTs) and AuNRs.^210^ As an imaging probe to localize specific diseases or tissues, SPNs can produce PA signals in the sites of interest, relying on simple accumulation via the retention effect or receptor binding on the cell surface. This strategy leads to the application of LN mapping,^210^ *in vivo* tumor-targeted imaging,^211^^–^^213^ cell tracking,^214^ and ROS mapping.^134^ For example, SPN with poly as cyclopentadithiophene-alt-benzothiadiazole showed a maximum NIR-I absorption at 660 nm and maximum PA signals at 690 nm, and it was used as PAI probes for lymphatic imaging.^210^ Another recently reported SPNs, which was fabricated into water-dispersed NPs, showed strong absorption in the NIR-II window and efficient cellular uptake, leading to effective accumulation in subcutaneous tumors and brain tumors.^212^

**Fig. 5:**
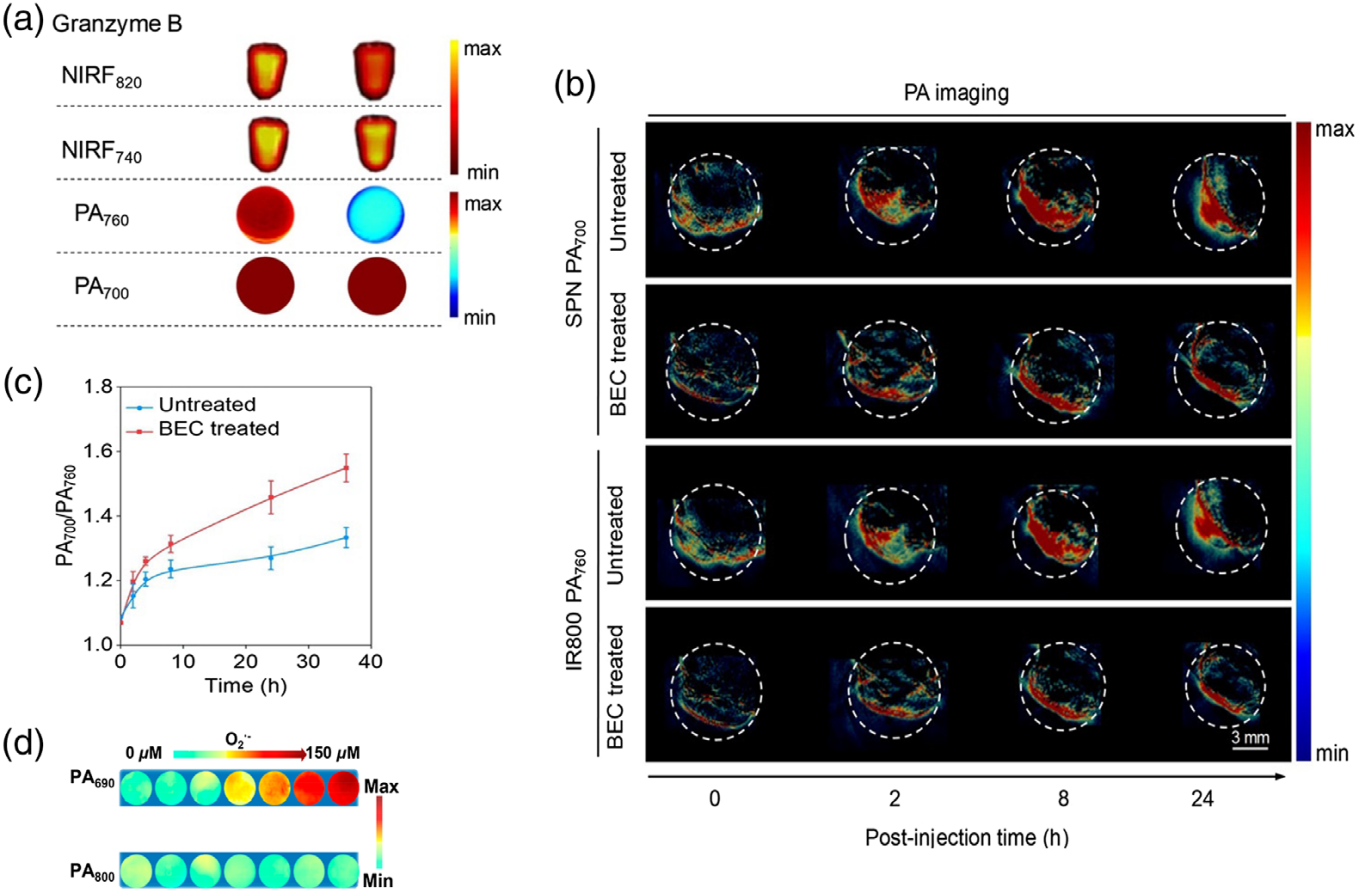
(a) Under 640- and 745-nm laser irradiation, fluorescence images of SPNP solution at the emission of 740 and 820 nm, respectively; PA images of SPNP solution at 760 and 700 nm with or without granzyme B. (b) After intravenous injection of SPNP, PA images of untreated and BEC-treated 4T1-tumor-bearing mice at different time points. (c) Quantification of PA intensity at 700 nm to that at 760 nm of tumors sites. The tumor-bearing mice were untreated or treated with BEC, followed by injection of SPNP. The ratio was a function of post-injection time of SPNP. (a), (c) Reprinted with permission from Ref. 104. (d) Under 690 and 800 nm, the changes of PA signals for RSPN (7  μg/mL of ORM) in different concentrations of ${O2}^{-}$ (0 to 150  μM). Reprinted with permission from Ref. 216. Copyright 2021 ACS.

Other than the simple accumulation of agents, SPNs also can be employed as activable PA probes with biomarker-triggered signals, which offer measurable and quantifiable real-time information on pathological status at the molecular level. Such smart PA probes have been used for *in vivo* imaging of a variety of biomarkers and biological metabolites in recent years, including abnormal pH,^215^ different ROS imaging (such as ${\mathrm{ONOO}}^{-}$ and ${\mathrm{CLO}}^{-}$ level,^134^^,^^210^ $O2^{•-}$ level^216^), and granzyme B.^104^ The reader can refer to Ref. 104 for more details on activatable SP nanoprobe (SPNP), which was reported as a biomarker to detect granzyme B in the tumors of living mice. Granzyme B can be considered to be a signature of immune activation due to elevated concentration in cytotoxic T lymphocytes. SPNP consisted of core SP conjugated with granzyme B-responsive and dye-labeled peptides (IR800) and can simultaneously show NIR fluorescence (NIRF) and PA signals. SPNP showed PA signals at 700 and 760 nm, corresponding to SP and IR800 absorption peaks, respectively. In the presence of granzyme B, PA signals decreased at 760 nm but remained unchanged at 700 nm [Fig. 5(a)]. The effectiveness of SPNP was evaluated with *in vivo* tumor-bearing mice model. It was shown that the PA signals gradually increased and reached a similar highest intensity level for both immune-activated mice and those normal mice at 700 nm laser excitation [Fig. 5(b)]. According to Fig. 5(c), PA700/PA760 was 1.2 times higher for immune-activated mice than those normal mice, suggesting that the granzyme B levels were higher in the tumors of living mice treated with an immunotherapeutic agent. Negligible cell cytotoxicity toward CD8+ T cells and 4TA cells even at a high concentration was observed, and the small average dynamic size (∼25  nm) made it an effective probe for *in vivo* imaging of immune activation associated with T lymphocytes.

**Fig. 6:**
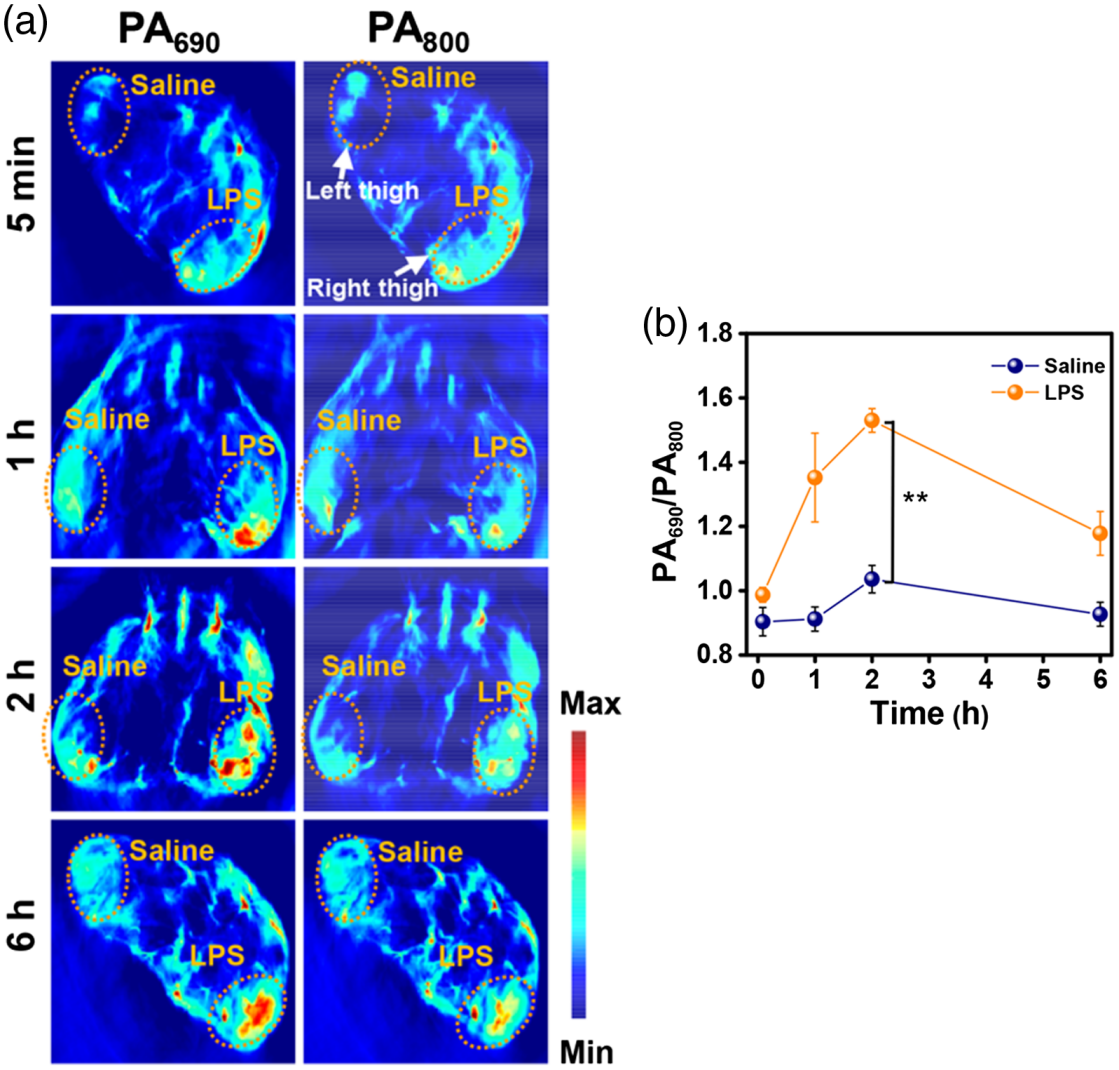
(a) PA images of LPS-stimulated mice model of acute inflammation. The left and right thighs (indicated by white arrows) were preinjected with saline and LPS, respectively, for 18 h, followed by subcutaneous injection with RSPN (n=3). (b) The ratio of PA intensity at 690 nm to that at 800 nm in regions injected with saline and LPS separately. Data are shown as mean ± standard deviation (n=3) (two-tailed Student’s t test, **p<0.01). (a), (b) Reprinted with permission from Ref. 216. Copyright 2021 ACS.

Another application explored was imaging of ${O2}^{•-}$ level within atherosclerotic lesions.^216^ A high level of ${O2}^{n-}$ could be produced in the vulnerable atherosclerotic lesions, resulting in the occurrence of inflammation. The proposed ratiometric semiconducting polymer nanoparticle (RSPN) was composed of an ${O2}^{•-}$-responsive molecule (ORM) and insensitive SP molecule (OIM), which performed as a responsive group for ${O2}^{•-}$ and internal PA reference, respectively. In the presence of ${O2}^{•-}$, ORM could enhance absorption at 690 nm due to intramolecular charge transfer, whereas the absorption at 800 nm remained nearly unchanged [Fig. 5(d)]. Taking the absorption peak of OIM at 800 nm as a reference, RSPN provided a reliable determination of $O_{2}$ by the changed ratios of PA signals (${\mathrm{PA}}_{690}/{\mathrm{PA}}_{800}$). The effectiveness of RSPN was verified in macrophages established lipopolysaccharide (LPS)-induced acute inflammation mice model. The corresponding thighs, which were pretreated with LPS and injected with RSPN, showed a stronger PA signal at 690 nm [Fig. 6(a)] and higher ${\mathrm{PA}}_{690}/{\mathrm{PA}}_{800}$ [Fig. 6(b)] than the opposite site pretreated with saline, indicating the effective assessment of the inflammation level of atherosclerosis. In addition, plaque-bearing mice were also employed and showed an enhanced PA signal in the aorta after injection of RSPN. This report demonstrated RSPN as a powerful tool for ${O2}^{•-}$ level imaging and vulnerability of atherosclerotic plaque monitoring. In addition to ${O2}^{•-}$ mapping, activatable probes based on organic semiconducting nanoprobes doped with bulky borane were also designed for *in vivo* ratiometric imaging of peroxynitrite (${\mathrm{ONOO}}^{-}$).^134^

### 3.5 Carbon-Based Probes

Other than the materials discussed in Secs. 3.1–3.4, carbon-based materials, such as CNTs,^139^^,^^217^^–^^219^ carbon nanodots,^220^^,^^221^ and carbon nanohorns,^222^ have been explored as PA agents. For example, ICG-enhanced single-walled CNT conjugated with Arg-Gly-Asp (RGD) peptide was designed to target $\alpha_{v}\beta_{3}$ integrins, which was related to the blood-vessel growth of tumor.^218^ Injection of this contrast agent to tumor-bearing mice showed specific tumor targeting, strong contrast, and high detection sensitivity in PA images. The disruption of atherosclerotic plaque results in myocardial infarction and stroke; therefore, a diagnostic method to detect the disease in advance is in great demand. CNTs that selectively targeted $\mathrm{Ly}\text{-}{6C}^{\mathrm{hi}}$ monocytes were used to image inflamed plaques in a mouse model.^217^ As $\mathrm{Ly}\text{-}{6C}^{\mathrm{hi}}$ monocytes produce foamy macrophages and both appear in arterial walls, agents that specifically target these immune cells can provide relevant information of plaques. The agents were evaluated in excised arteries in phantoms using PAI, and the results demonstrated that PAI can reliably detect atherosclerotic plaques. Wu et al.^220^ also developed carbon-based nanomaterials for PAI. Porphyrin-implanted carbon nanodots (PNDs) were prepared and showed good stability, strong NIR absorption, and biocompatibility. After conjugating with cetuximab, the PNDs were further used to accurately target cancer cells that overexpressed EGFR. The cell targeting ability was verified in mice bearing MDA-MB-231 breast cancer. In addition, carbon nanodots were also employed by Lee et al.^221^ These carbon nanodots were doped with nitrogen, resulting in strong absorption in the NIR region and excellent performance for imaging of SLN. In addition, they can be used as PTT agents for cancer ablation. Other than CNTs and carbon nanodots, carbon nanohorns were also employed as PA probes due to their large specific surface area, low toxicity, and unique physicochemical properties. Via surface engineering, Li et al.^222^ developed a carbon nanohorn-polyglycerol-gold nanomaterial, which was loaded with the chemotherapeutic drug doxorubicin (DOX). The resulting nanohybrid (DOX@CNH-PG-Au) presented steady accumulation at 4T1 tumors after intravenous administration, leading to clear observation of the tumor *in vivo* with PAI.

### 3.6 Proteins

In addition, genetically encoded proteins were considered to be an efficient PA probe for imaging of cellular dynamics,^223^ ${\mathrm{Ca}}^{2+}$,^224^ whole-brain neuronal activity,^74^ protein–protein interactions in mouse tumors,^225^ and tumor growth and metastasis.^226^ Based on a pair of fluorescent proteins, miRFP670 and iRFP720, an NIR Forster resonance energy transfer (FRET) biosensor was reported to monitor apoptosis in single cells and in mouse tumors *in vivo*.^223^ miRFP670 donor and iRFP720 acceptor were linked by the -DEVD- linker, showing high PA signals under the excitation of the laser. During apoptosis, caspase-3 cleaved the linker, resulting in a significantly lower PA signal being produced by the biosensor. By detecting the PA signal difference, apoptosis in individual HeLa cells was observed. The caspase-3 activities were also monitored in a mouse ear tumor and brain tumor, demonstrating that this FRET activatable biosensor had great potential in imaging dynamic biological processes. Gottschalk et al.^74^ presented that neuronal activity of the mouse’s whole brain could be detected under a single laser irradiation. After current stimulation at the right hind paw, PA signals changed rapidly in the activated brain area, indicating somatosensory-evoked quick calcium transients. The responses of GCaMP6f and GCaMP6s were also compared under electrical stimulus of the right and left hind paws, respectively. GCaMP6s signals reached a peak at nearly the same time as GCaMP6f signals but showed a slower decay. The outcomes indicated that PAI can be used as a sensitive tool for neuroimaging. Encoded proteins that were used as PA probes for other applications are discussed in Sec. 4.

## 4 Activatable, Photoswitching, and Temperature-Dependent Photoacoustic Probes

**Fig. 7:**
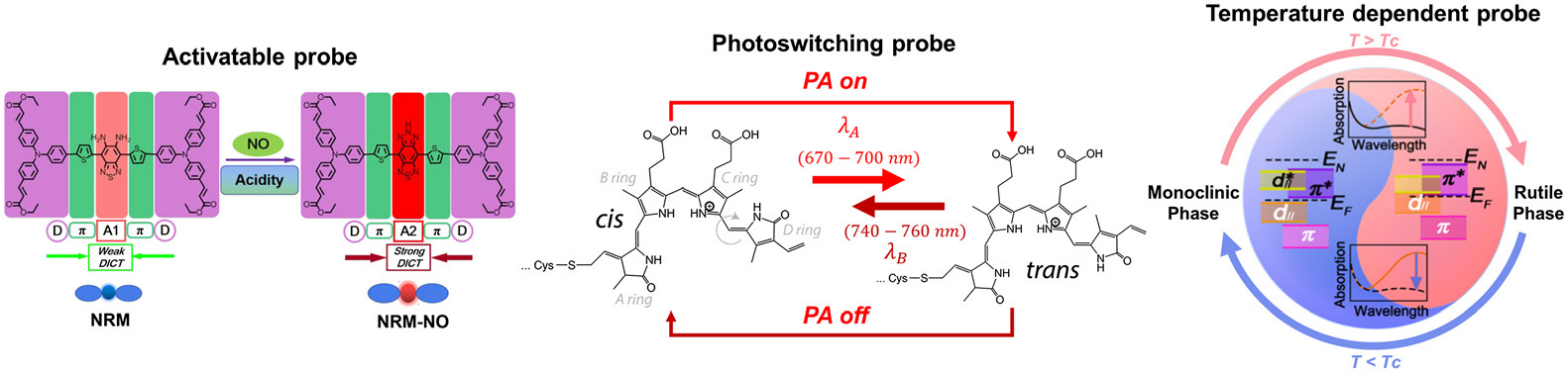
Activation mechanisms of biomarker/ microenvironment activatable probes illustrated with the responsive mechanism of NRM to NRM-NO in the presence of NO or acidity. Adapted with permission from Ref. 227. Copyright 2019 ACS. Reversibly photoswitching probes can switch between cis (off) and trans (on) states as a result of specific wavelength irradiation. Adapted with permission from Ref. 228. Copyright 2019 ACS. Temperature activated probes exhibit varied absorption spectra as the surrounding temperature changes. Reprinted with permission from Ref. 229. Copyright 2022 ACS.

The PA probes reported above may use retention effects or receptor binding to localize and target specific diseases and tissues. However, background and endogenous PA signals frequently interfere with signals induced at the site of interest, leading to low contrast and possibly false diagnostic outcomes. This has motivated the research for probes that can be triggered “on” or “off” for distinct contrast as opposed to passive contrast agents. Controllable probes also allow for greater contrast resolution, especially when imaging at greater depths, and can be paired with conjugates as with other probes for molecular imaging. Such probes have been divided into three categories based on the trigger: a biomarker, specific light stimulation, or temperature changes (as shown in Fig. 7). The functioning mechanism of these probes is often similar as they require the trigger to ultimately induce a change in the probe’s molecular form, the absorption spectrum, and the subsequently detected PA signal.

### 4.1 Activatable Probes

The most common type is activable probes,^230^ which only respond to specific biomarkers, such as low pH in tumor microenvironments. Activable PA probes have been used for *in vivo* imaging of a variety of biomarkers and biological metabolites and therefore imaging of aberrant tissues in recent years. Usually, the activatable nanoprobes are designed to be activated by some single-factor stimulation, such as granzyme B,^104^ different ROS (such as ${\mathrm{ONOO}}^{-}$,^134^ ${O2}^{•-}$,^216^ NO^231^), low pH,^215^^,^^232^ ${\mathrm{Cu}}^{2+}$,^233^ and caspase-3.^223^ In the presence of the stimulation biomarker, the absorption spectrum changes, leading to the alteration of PA signals in the region with abundant biomarkers. Previously, a series of activatable probes (RPS1-RPS4) for *in vivo* tracing of ${\mathrm{Cu}}^{2+}$ was developed by Wang et al.^233^ The PA signal intensity from the probes was tuned by molecular structure modification. For example, after the introduction of electron-donating group (N,N-dimethylaniline), the resulting probe (RPS1) showed enhanced PA intensity, high stability, and fast response to ${\mathrm{Cu}}^{2+}$. As the best probe in the series, RPS1 could cross the blood–brain barrier (BBB) and allowed for *in vivo* visualization of ${\mathrm{Cu}}^{2+}$ in the mouse brain via PAI. As the abundance of ${\mathrm{Cu}}^{2+}$ in the brain is closely related to Alzheimer’s disease (AD), AD mice and age-matched normal mice were used for assessing the probe performance *in vivo*. There were weak PA signals shown in the brains of normal mice, whereas strong PA signals were observed in the brains of AD mice, especially in the cortex region. The outcomes indicated that RPS1 can be an effective PA probe to cross the BBB and visualize ${\mathrm{Cu}}^{2+}$ *in vivo*.

**Fig. 8:**
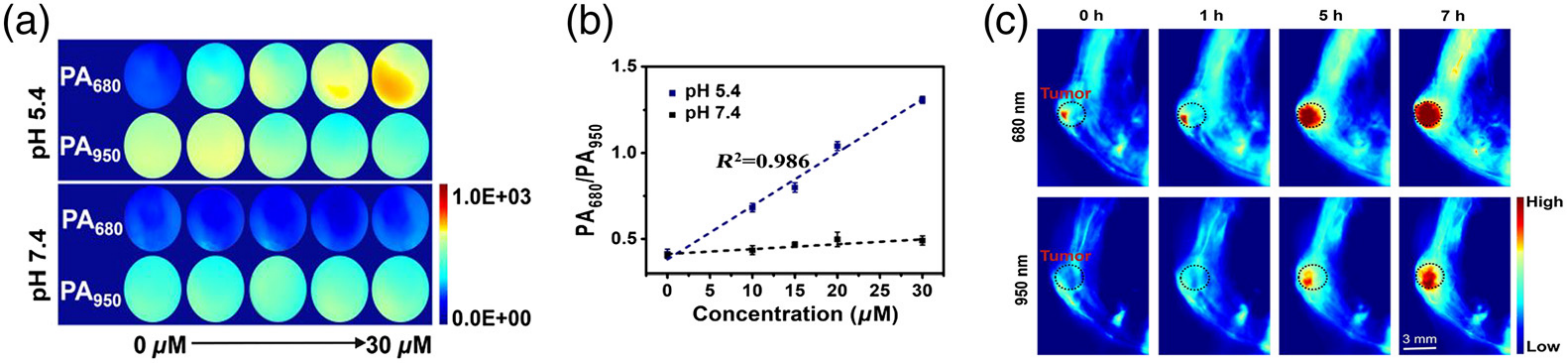
(a) PA images of dual-activated theranostic nanoprobe (DATN) in response to different concentrations of NO. (b) PA680/PA950 of DATN in different concentrations of NO. (c) Tumor-bearing mice were injected with DATN (10  mg/kg) and corresponding PA images of tumor sites were taken at 680 and 950 nm at indicated times (0, 1, 5, 7 h). Reprinted with permission from Ref. 227. Copyright 2019 ACS.

However, having a single stimulation factor can restrict specificity between diseased and normal tissues. Therefore, a few nanoprobes were presented to be responsive to dual stimulation. Teng et al. developed a “dual-key-one-lock” nanoprobe for cancer imaging, which responded to both nitric oxide (NO) and acidity.^227^ An NO-responsive molecule (NRM) and NO-insensitive molecule were incorporated into a nanoparticle and showed an increased PA signal at 680 nm and a nearly constant signal at 950 nm under elevated levels of NO and low pH [Fig. 8(a)]. Taking the absorbance at 950 nm as reference, the absorbance ratio $A_{680}/A_{950}$ can be utilized, and thus the nanoparticle was employed as a ratiometric probe. As shown in Fig. 8(b), under low pH condition, the ratio of PA signals at 680 nm to PA signals at 950 nm (${\mathrm{PA}}_{680}/{\mathrm{PA}}_{950}$) increased significantly. Tumor-bearing mice were injected with NPs for assessing the *in vivo* PAI ability. The PA signal in the tumor region showed a quicker increase and higher level at 680 nm than the signal at 950 nm over time, indicating that the probe had better responsivity at 680 nm to NO and low pH [Fig. 8(c)]. These results showed that the agent is promising for high specificity tumor imaging with good biocompatibility. A ratiometric PA probe (RSPN) was also introduced for *in vivo* imaging of reactive oxygen species.^210^ In the presence of ${\mathrm{ONOO}}^{-}$ and ${\mathrm{CLO}}^{-}$, the PA signal peak at 820 nm almost disappeared, whereas the peak at 700 nm remained nearly unchanged. *In vivo* PAI was done in a murine model of acute edema. Zymosan, which can simulate the generation of ROS, was injected into the thigh of living mice, followed by the injection of the contrast agents. The post-injection images showed that the ratio of the PA signal at 700 nm to that at 820 nm increased significantly, which indicated that RSPN can effectively detect ROS production in real time.

Gold core nanorods coated with silver have been widely explored due to their flexible switch between the “on” and “off” states. Bare AuNRs have NIR absorption, which is blueshifted after being coated with silver, resulting in a lower PA signal. When the shell is reacted with the ferricyanide solution or oxidized by the reactive oxygen and nitrogen species (RONS), the silver shell was etched and the NIR resonance was restored. The recovered PA signal offered localization of Ag+ ions. Such smart probes have been explored in many applications, including antibacterial treatment,^234^ tracking RONS with high sensitivity,^158^ and monitoring multidrug-resistant *Staphylococcus* *aureus* infection.^235^ Such probes respond to biochemical cues via endogenous or exogenous activation, whereas other probes rely on enhanced accumulation to generate strong PA contrast.

### 4.2 Photoswitching Probes

A second, more recent alternative is the use of photoswitching (or photochromic) probes that can change their absorption and other photophysical properties when excited with light of a certain bandwidth. The switch occurs in the form of a red- or blueshift of the agent’s original absorption band to a second band. Probes that can recover from the change indefinitely, i.e., reversibly switching probes, are more desirable due to their repeatable and sustained use. Such probes are typically proteins that have either an intrinsic or engineered capability to induce these changes. Further explorations of nonlinear physics and chemical mechanisms have been reviewed elsewhere.^228^^,^^236^

**Fig. 9:**
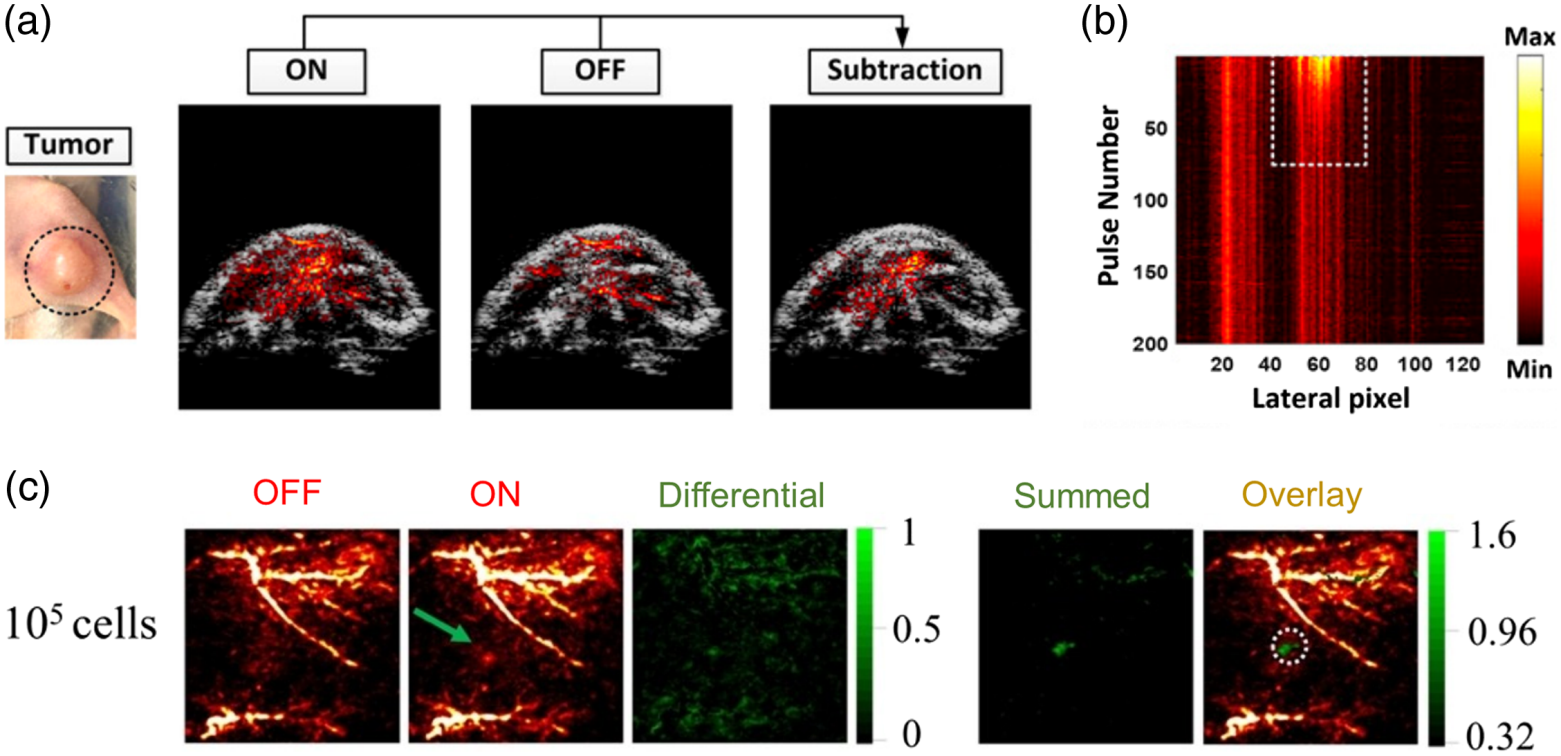
(a) PAI of mouse tumor using the bacterial-based photoswitching probe (mDrBphP-PCMm/F469W) overlaid with US. (b) MAP of the bscan area in (a) against time. The persistent signals indicate endogenous PA signals, and the decaying signal bounded by the white dashed rectangle shows the *E. coli* accumulation. (a), (b) Reprinted from Ref. 239. Copyright 2022 National Academy of Sciences. (c) Labeled HeLa cell tracking with endogenous signals (off), probe switched on, a differential of the two, a subsequent cumulative addition of differentials from 10 cycles of switching (summed), and a final overlay with the initial endogenous signal and the predetermined cell location circled in white dashed lines. Reprinted with permission from Ref. 240. Copyright 2022 Wiley-VCH GmBH.

A key advantage of photoswitching probes is the ability to effectively modulate the PA contrast by turning the probe “on” and “off” rapidly. This rapid change with the knowledge of the incoming excitation light leads to several variations of lock-in detection or signal unmixing in which the alternating signal can be temporally extracted from the unchanging background of either tissue or nearby endogenous chromophores. Similar to their application in super-resolution fluorescence imaging,^237^ these probes can also be used to enhance contrast^225^ and improve imaging resolution in optical resolution photoacoustic microscopy^226^^,^^238^ and mitigate photobleaching. As pointed out by Mishra et al.,^228^ in their extensive review, the next step in such agents’ progression is the ability to target specific components. This was earlier demonstrated by Li et al.,^225^ and more recently shown by Gao et al.,^239^ with their phytochrome-based reporter protein coupled to the tumor targeting bacteria, *E. coli*, to achieve targeted *in vivo* mouse imaging [Fig. 9(a)]. Figure 9(b) shows the maximum amplitude projection (MAP), in which the decaying signal from the probe after it is switched off is visible.

Typically, reversibly switchable probes are based on the fluorescent protein bacteriophytochrome, allowing for absorption in the NIR region. The progressive development of such probes and parallel versions of them has been reviewed extensively.^241^ Liu et al.,^240^ however, recently developed upconverting nanoprobes that were able to reversibly switch states with far-red and NIR excitation. These particles were demonstrated via labeled HeLa cells injected in mice and shown to have a 0.05-nM detection limit using differential imaging. They were also able to detect as little as $10^{5}$ cells with 10 switching cycles as shown in Fig. 9(c) or $10^{4}$ cells with 22 cycles.

Mishra et al.^238^ were able to demonstrate whole-animal imaging with two reversibly switching probes, *Re*BphP-PCM and *Rp*BphP1-PCM, based on the truncated forms of bacteriophytochromes *Rhizobium etli.* and *Rhodopseudomonas* *palustris*, respectively. The two probes coupled with a previously developed *Dr*BphP-PCM probe^225^ provided different photoswitching speeds that can be exploited for time-resolved multiplexed imaging. Temporal unmixing was used to successfully differentiate *in vivo* Jurkat T lymphocytes expressing *Re*BphP-PCM or *Dr*BphP1-PCM and *E. coli* expressing *Re*BphP-PCM injected in the same mouse model. Among other experiments, the group was able to identify as few as 500  cells/μL of Jurkat T lymphocytes coexpressing *Re*BphP-PCM and green fluorescent protein.

### 4.3 Temperature Dependent Probes

Another mechanism for the controlled activation of a probe is the use of surrounding temperature changes to switch absorption properties. The temperature change can either occur within the host or can be changed with laser irradiation. Li et al.,^229^ developed a reversible PEG-coated tungsten-doped vanadium dioxide (W-VO2@PEG) probe that showed an increased signal when surrounding temperatures were increased from 35°C to 45°C and had <10% signal fluctuation after 10 temperature cycles. EMT6 tumor-bearing mice were injected with a solution containing the probes and then irradiated with a 980-nm laser to increase the temperature. After a few minutes of irradiation, the PA signals from the tumor site were more pronounced. This probe was reported to alleviate the current issues of nonreversible absorption changes or probes with a trigger temperature that is high enough to potentially damage the surrounding tissue.

Yang et al.^242^ also developed temperature-dependent probes based on pyrrolopyrrole cyanine derivatives (PPC NPs) that are nonspecific. These probes take advantage of fluorophore’s reversible switching between monomeric and J-aggregate states to induce light absorption changes. When heated from 25°C to 45°C for 10 min, the ${\mathrm{PPC}}_{15}$ probes absorb 3.5 times less light at 770 nm but can subsequently recover as the temperature returns to 25°C. The probes showed the same trend in *in vivo* mouse tumor imaging and showed potential for PTT when a probe with a different degree of polymerization, ${\mathrm{PPC}}_{2}$, was used. Such probes enable a completely independent mechanism of activation that is not reliant on light excitation or biomarkers.

## 5 Molecular Photoacoustic Imaging

Label-free PAI falls short when more specific or targeted imaging is required to study molecular events or view trace concentrations of early-disease biomarkers, as mentioned in Sec. 2. These limitations may result from limited optical or acoustic resolution or from the subject of study being overshadowed by signals from the more abundant but uninformative surroundings. Though the use of contrast agents significantly increases the sensitivity of PA detection, a new set of challenges arise regarding the desired specificity, the most important being targeting efficacy to facilitate molecular imaging. Other considerations include accumulation in the targeted site, circulation time in the body, PA generation efficiency, and maximum achievable imaging depth. The selection processes and design considerations for targeted and activatable probes have been recently reviewed.^230^^,^^243^^,^^244^ In this section, we will review some of the recent molecular PAI applications with PA probes.

### 5.1 Sentinel Lymph Node Detection

**Fig. 10:**
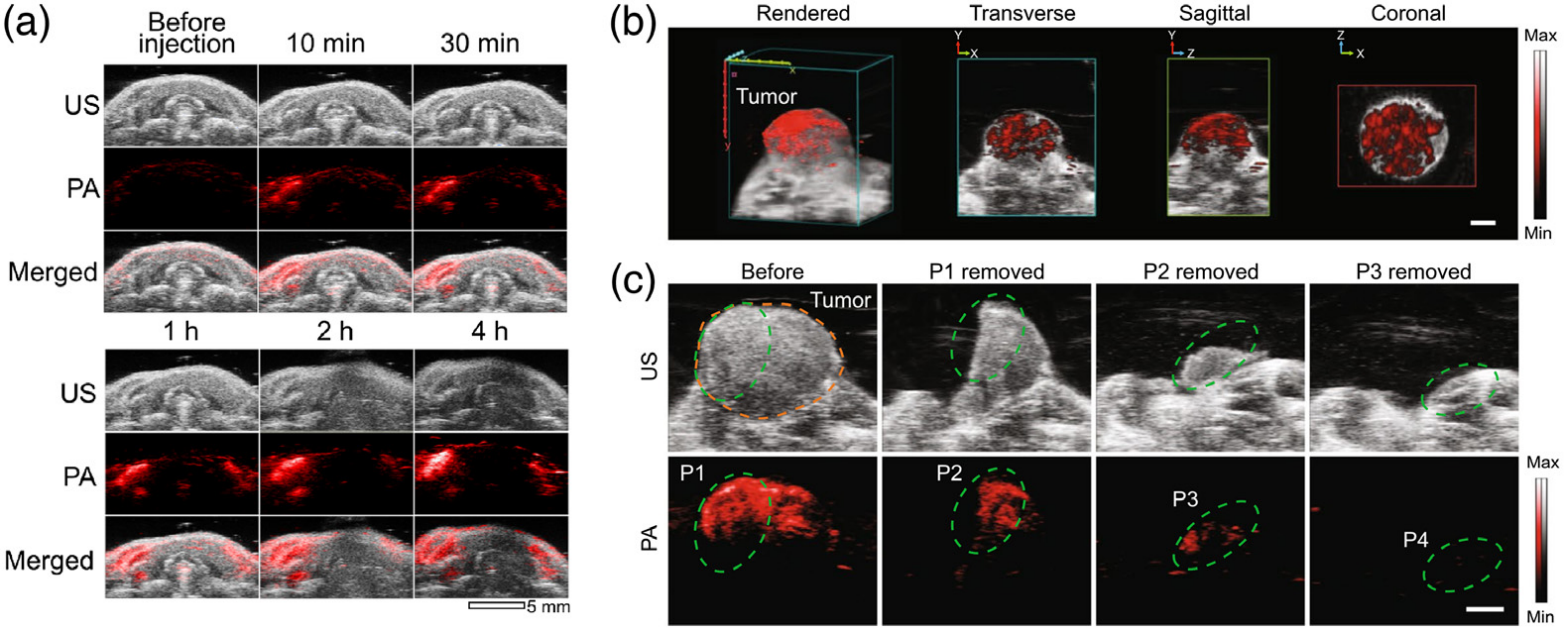
(a) Mouse cervical LNs imaged with PA using OVA-GC-AuNPs and overlaid with US. Reprinted from Ref. 247. (b) A 3D reconstructed tumor mass using the cRGD-MNPs overlaid with US. This reconstruction was used to guide the subsequent resection. (c) The resection of the tumor mass in three steps (P1-P4) and verification with PAI in the last step of no tumor remaining. (b), (c) Reprinted from Ref. 248.

Knowing the status of SLNs has prognostic value in cancer treatment as these nodes are the first drainage route for spreading of the cancer cells. SLN biopsy is a “gold standard” for assessing the SLN status, and various techniques (including PAI) are used in combination with contrast agents.^245^ Initially, multimodal metastatic LN targeting was demonstrated using AuNPs coupled with anti-EGFR and anit-RG16 monoclonal antibodies^246^ for metastases of size $2.6\times 10^{-3}\;{\mathrm{mm}}^{3}$ or larger. More recently, glycol-chitosan-coated gold nanoparticles (GC-AuNPs) were used for targeted imaging of cervical LNs as shown in Fig. 10(a). These particles also proved effective for tumor antigen delivery when attached to ovalbumin (OVA) epitope.^247^ Another approach for more specific imaging of metastatic SLNs while avoiding normal or inflamed LNs was shown in the form of dual-targeting probes.^249^ The 5K-HA-HPPSs probes target CD44 and scavenger receptor class B1 (SR-B1), both of which are expressed in breast cancer. The probes were tested on 4T1 tumor-bearing mice, and the PA signal was found to accumulate within the metastatic tumors but only on the periphery of normal or inflamed LNs.

### 5.2 Brain Diseases

The detection of excessive beta-amyloid deposits is crucial for detecting Alzheimer’s disease. Multimodal *in vivo-*targeted imaging of beta-amyloid fibrils was demonstrated using CRANAD-2 in real time using arcAβ mice.^250^ Further, whole-brain targeted imaging of tau deposits, also a factor in diagnosing Alzheimer’s and dementia, was shown using pyridinyl-butadienyl-benzothiazole derivative PBB5.^251^ An extensive review of molecular imaging of various brain diseases using PAI was recently published.^252^

### 5.3 Cancers and Tumor Microenvironments

Abnormal tumor microenvironmental factors, such as low extracellular pH, hypoxia, and upregulated expression of tumor-related proteases, can be taken as cancer signatures.^207^ MMPs are vital biomarkers for tumorigenesis, metastasis, and angiogenesis of cancers, and MMP-2 is one of the most important MMPs as it is overexpressed in most solid tumors. Clinical diagnosis and therapeutic evaluation of cancers are related to the MMP-2 activity *in vivo*. In addition, the overexpression of MMPs was well correlated with abnormal pH *in vivo*, and their combined effects greatly influence the heterogeneous invasion of malignant tumors. As MMP-2 can be secreted by cancer cells, murine breast carcinoma 4T1 cells were chosen as MMP-2-positive cancer cells due to the high level of MMP-2 expression, whereas 3T3 cells were taken as a negative control.

On the assessment of tumor margins, cyclic Arg-Gly-Asp melanin nanoparticles (cRGD-MNPs) were used to target MDA-MB-231 tumors in mice.^248^ The probes showed better signals than using melanin-based nanoparticles (MNPs) alone and were used to reconstruct a 3D profile of the tumor as shown in Fig. 10(b) overlaid with US images. The tumor was then partially resected twice, and the PA signals from the probes were used to verify the decreasing size of the tumor as shown in Fig. 10(c). Finally, upon excision of the remaining tumor, PAI and histological analyses confirmed that no tumor remained behind. The maximum depth of detection was shown to be 5 mm. This study presented a very promising prospect for the identification of residual malignant tissue, guided surgery, and eventual mitigation of resection. An extensive tutorial review by Zhao et al.^243^ goes over common cancer biomarkers used for molecular imaging. Other exogenous probes specific to tumor-targeting are detailed in another recent review.^85^

### 5.4 Calcium Imaging

A particular application of interest in the field of neuroscience is calcium imaging as it enables neuronal activation mapping on a larger scale than current electrophysiological techniques. As opposed to electrodes that record electric pulses, exogenous probes target the neurons using markers such as calcium and provide enhanced imaging. Recently, Shemetov et al.^253^ developed a fully NIR genetically encoded calcium indicator (iGECI). The probe was able to characterize neuronal activity and hemodynamic activity, i.e., increased blood oxygen in the active regions of the brain, simultaneously. This was shown *in vivo* using a mouse hybrid in which the hindlimb was electrically stimulated. The corresponding vasculature images obtained using PAI showed significant increases in blood oxygenation of the target cortical regional in addition to the ${\mathrm{Ca}}^{2+}$ signal.

Abnormal ${\mathrm{Ca}}^{2+}$ expression has also been correlated to tumorigenesis. As such, chlorophosphonazo III (CPZ III) was explored for labeling intracellular ${\mathrm{Ca}}^{2+}$ in 3D tumorspheres cell cultures.^254^ Although demonstrated *ex vivo*, the probe functions with NIR-I excitation, which allows for deeper imaging. In addition, the probe absorbance varied with the surrounding pH, which may allow for pH-sensitive use. Finally, Mishra et al.^224^ studied the development of a reversibly photoswitching ${\mathrm{Ca}}^{2+}$ sensors based on GCaMP5G (rsGCaMP1.1 and rsGCaMP1.4-eR). The probe was triggered using 405 and 488 nm light and was demonstrated in phantoms as well as HeLa cells expressing rsGCaMP1.1 subcutaneously implanted onto a mouse. Such probes further enhance nanoscale imaging of ions with the contrast advantage that photoswitching provides.

### 5.5 Other Applications

An emerging and exciting direction of research within PAI is the development of microrobots. Such probes offer greater levels of control over targeting compared with the techniques discussed above. Wu et al.^255^ developed a PACT-guided microbotic system that comprises magnesium-based micromotors transported inside micromotor capsules. These capsules were engineered such that, upon continuous-wave NIR irradiation, the micromotors would be released and begin their own gas propulsion toward the diseased area. Remarkably, the probes could be administered orally, were stable in the acidic environment of the stomach and GI tract unless activated, and were biocompatible. The probes also showed increased retention once the micromotors were released from their capsules based on histological analyses of the intestines. This direction of probe development provides a parallel method for the delivery of drugs or the enhancement of contrast in areas of interest.

As in the previous section on calcium imaging, voltage mapping also seeks to map neuronal activity in the brain by capturing the differences in voltage as opposed to an ion. Although this is done on a macroscale with electrodes, Rao et al. demonstrated that it is possible to map these signals using PAI and the voltage sensor dipicrylamine (DPA).^256^ This was demonstrated using HEK-293 cell membranes with varying resting potential changes and cross correlated with spectrophotometric analyses of varying $K^{+}$ concentrations. Although the mechanism of imaging was not considered to be fast enough for real-time imaging, *in vivo* acquisitions of mouse brain response after electrical stimulation and induced epilepsy showed distinct voltage response signals. Critically, these signals could be differentiated from hemodynamic signals.

Finally, targeted potassium probes were developed using ionophore-based solvatochromic dye-based K+-sensing nanoparticle (SDKNP).^257^ The probes were tested on tumor-bearing mice and showed remarkable PA signal difference due to the higher concentration of $K^{+}$ in tumors. Further, the group noticed slightly higher concentrations of $K^{+}$ inside the tumor’s core as opposed to the tumor’s periphery. The extraction of the PA signal, however, requires spectral unmixing and PAI with several wavelengths.

## 6 Multimodal Imaging

Every imaging modality has their advantages and disadvantages. Sometimes, a combination of multiple imaging modalities allows us to overcome the limitations of an individual modality and provide more reliable and precise detection of disease sites. Thus, multimodal imaging has great potential for obtaining high-quality images of the site of interest. With more and more multimodal imaging systems being developed, there is also a need for contrast agents that will work on these multimodal systems simultaneously. Sometimes, two separate contrast agents (each working for a different imaging system) are used concurrently to facilitate multimodal imaging. However, materials are also designed such that a single multimodal agent can work for different imaging systems simultaneously. PAI has been combined with various other imaging modalities, such as MRI,^180^^,^^258^^–^^260^ US imaging,^100^^,^^101^^,^^261^ fluorescence,^262^^–^^265^ PET,^206^ surface-enhanced Raman scattering (SERS),^172^^,^^232^^,^^266^ OCT,^267^^,^^268^ and two-photon microscopy^269^. At the same time, multimodal contrast agents have also been developed to work in sync with these multimodal imaging systems.

### 6.1 Photoacoustic-Fluorescence Probes

The molar coefficient and the nonradiative QY are two important factors for generating strong PA signals.^91^ Thus, fluorescent materials with low fluorescence QY hold great potential as PAI agents. In recent years, different contrast agents have been designed as dual fluorescence and PA probes for detecting RONS, such as ${\mathrm{ONOO}}^{-}$,^264^ identification of breast ductal carcinoma,^172^^,^^184^ tumor-related receptor imaging,^198^ tumor imaging,^265^^,^^270^ monitoring enzyme activity,^232^ visualization of GI tract,^263^ and imaging of immune activation.^104^ For example, a molecular activatable probe was designed for *in vivo* fluorescence and PA dual-modal imaging of ${\mathrm{ONOO}}^{-}$, which is a highly reactive RONS and leads to mitochondria malfunction and cellular apoptosis in tumor sites.^264^ The probe (${\mathrm{CySO}}_{3}{\mathrm{CF}}_{3}$) was composed of a hemicyanine dye caged with a trifluoromethyl ketone moiety, having high detection accuracy, specific targeting to ${\mathrm{ONOO}}^{-}$, and adaptability to acid tumor microenvironments. ${\mathrm{CySO}}_{3}{\mathrm{CF}}_{3}$ was nonfluorescent in the caged state due to the zwitterionic structure of hemicyanine dye, but it converted into the uncaged molecule (${\mathrm{CySO}}_{3}\mathrm{OH}$) through a series of oxidation–elimination reactions as a turn-on response toward ${\mathrm{ONOO}}^{-}$. In the presence of ${\mathrm{ONOO}}^{-}$, the probe not only is fluorescent but also has redshifted NIR absorption at 675 nm. Subcutaneous 4T1 xenograft tumor-bearing living mice were used to perform *in vivo* fluorescence and PAI to monitor ${\mathrm{ONOO}}^{-}$. After the 680-nm-laser irradiation, the fluorescence and PA signals increased gradually in the tumor and reached their maxima after 3-h of injection.

In addition, another fluorescence/PA dual-modal probe was demonstrated by Li et al.^270^ As the organic NIR-II probe for fluorescence imaging always possesses donor–acceptor–donor structure and takes benzo[1,2-c:4,5-c0]-bis ([1,2,5] thiadiazole) (BBTD) as the core, it suffers from low QY. To enhance the QY, [1,2,5] thiadiazolo [3,4-f] benzotriazole (TBZ) was used as the acceptor, followed by D-A-D structure synthesis and nanoparticle formulation (BTB NPs). The contrast-enhanced ability of BTB NPs was verified with *in vivo* animal vasculature dual-model imaging, and it also could be used in molecular imaging with the Arg-Gly-Asp (RGD) peptide loaded on the NP surface. The synthesized BTB-RGD NPs were examined in nude mice model with inoculated 143B osteosarcoma cells and PC3 prostate carcinoma cells. The fluorescence image of the tumor sites presented a higher intensity for the BTB-RGD group compared with the BTB group or control group. Similarly, the tumor site of the BTB-RGD group showed a higher PA signal intensity than the other groups. The results demonstrated that BTB-RGD accumulated specifically at tumor sites and is an effective tumor-targeting dual-modal contrast agent. Ma et al.^263^ proposed an NIR-II fluorescence and PA probe for the detection of GI peristalsis disorder. The PDA polymer coated downconversion nanoparticles (DCNPs) showed effective accumulation in the small intestine, leading to deep tissue intestine imaging via both fluorescence imaging and PAI.

### 6.2 Photoacoustic-Ultrasound Probes

Both PAI and US imaging use similar signal receiving mechanisms which greatly facilitates the integration of PAI into existing clinical US systems. Therefore, in the last decade, several efforts have been made to make dual-modal clinical ultrasound and PAI systems.^72^^,^^271^^,^^272^ US imaging employs safe sound waves and can thus provide real-time imaging without any health hazard. However, US images often suffer from low contrast, especially for soft tissues. Microbubbles and nanobubbles are well-known US contrast agents.^273^ Some of them are also FDA approved for clinical use.^274^ Hence, efforts have been made to combine micro/nanobubbles with optical agents, so they can be used for dual-modal US/PA imaging.^261^^,^^275^ For example, Zhao et al.^275^ reported a dual-modal contrast agent that was composed of microbubbles loaded with vascular epithelial growth factor (VEGF) antibodies and fluorescent dyes. As the expression level of VEGF in the synovial tissues of rheumatoid arthritis (RA) patients is very high, VEGF can be regarded as a specific biomarker for the RA disease. In this study, the VEGF-targeted microbubbles were employed to detect the expression level of VEGF in the inflammatory tissues and therefore assess RA. Dual-modal US/PA imaging was performed for the inflamed paws of rats with arthritis and showed enhanced PA signals in inflamed sites as well as contrast-enhanced US images.

**Fig. 11:**
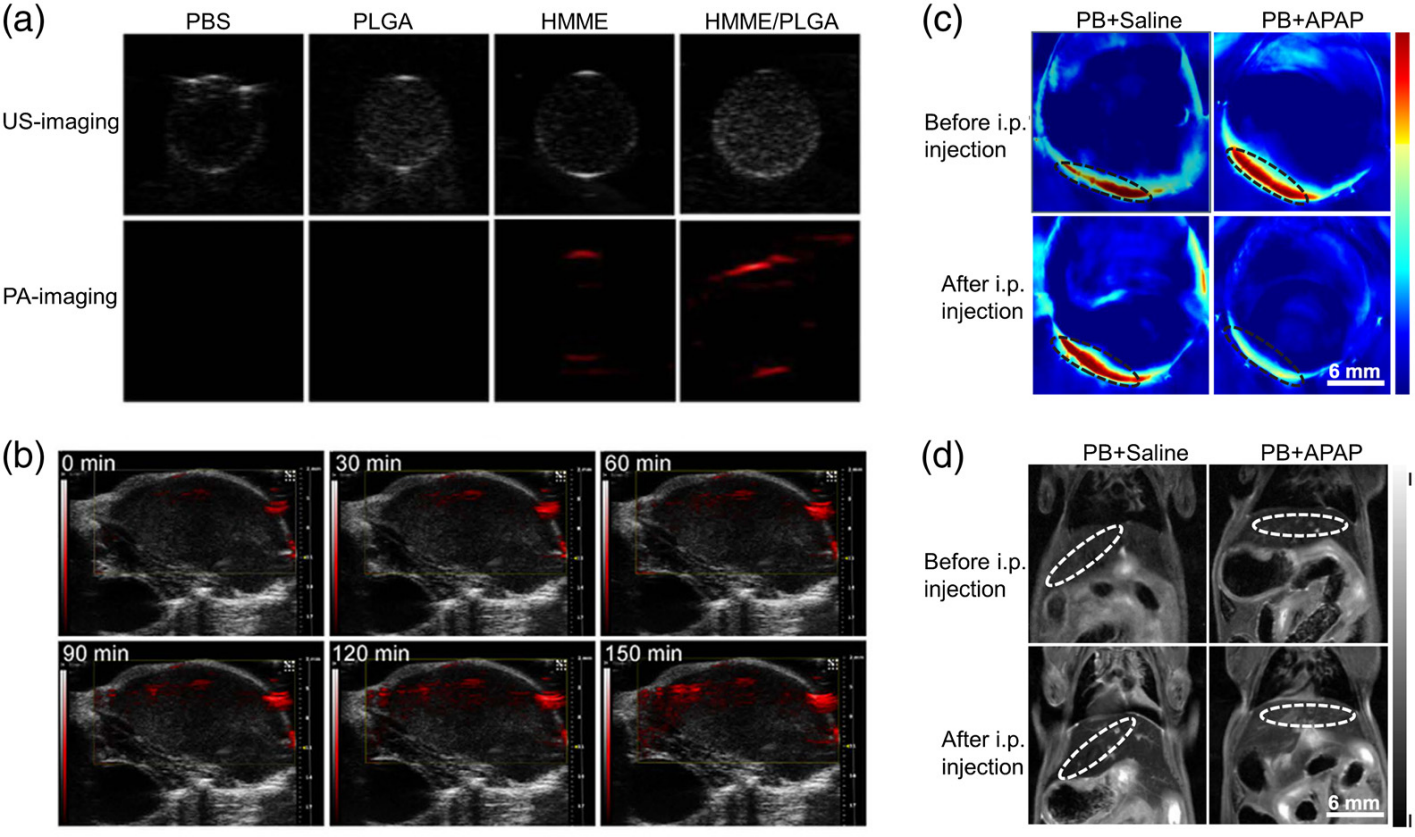
(a) At the same concentrations (0.50  mg/ml), *in vitro* US/PA imaging of four types of samples: PBS, PLGA, HMME, HMME/PLGA. (b) *In vivo* PA images of tumor tissue at 0, 30, 60, 90, 120, 150 min, respectively, after intravenous injection of HMME/PLGA microcapsules (1.5  mg/ml). (a), (b) Reprinted with permission from Ref. 277. (c) PAI images of mice injected with PB and saline or APAP. (d) MRI images of mice injected with PB and saline or APAP. (c), (d) Reprinted with permission from Ref. 278. Copyright 2020 ACS.

Other recent work includes nanocomposites that combined mesoporous ${\mathrm{TiO}}_{2}$ nanoparticles (${\mathrm{mTiO}}_{2}s$) with polypyrrole (${\mathrm{mTiO}}_{2}@\mathrm{PPY}$) for US/PA imaging contrast agent.^276^ Both *in vitro* and *in vivo* experiments showed that ${\mathrm{mTiO}}_{2}@\mathrm{PPY}$ had a strong US signal, and the signal intensity increased as the concentration of ${\mathrm{mTiO}}_{2}@\mathrm{PPY}$ increased. ${\mathrm{mTiO}}_{2}@\mathrm{PPY}$ also generated a PA signal in the tumor region of tumor-bearing mice, whereas no PA signal appeared in the mice injected with saline (control). These outcomes verified the dual-modal imaging properties of ${\mathrm{mTiO}}_{2}@\mathrm{PPY}$, which can also be used as contrast agents for imaging-guided therapy. In addition, hemotoporphyrin monomethyl ether (HMME)-loaded poly (lactic-co-glycolic acid) microcapsules (HMME/PLGA) were designed as dual-modal US/PA contrast agents that not only enhanced US imaging but also generated a good PA signal in the tumor region.^277^ At the same concentration (0.5  mg/ml), HMME/PLGA microcapsules exhibited the strongest US signals and PA signals, whereas PLGA showed nearly no PA signals and enhanced US signals compared with the phosphate buffered saline (PBS) group [Fig. 11(a)]. After intravenous injection of HMME/PLGA, the PA signal intensity increased gradually at the tumor region, which is shown in Fig. 11(b). These outcomes indicated that HMME/PLGA have great potential to be dual-modal contrast agents for enhanced US imaging and tumor-targeted PAI.

### 6.3 Photoacoustic-MRI Probes

MRI is also combined with PAI for complimentary contrast for imaging. MRI provides T1-weighted/T2-weighted/proton density contrast, whereas PAI provides optical absorption contrast. This complimentary contrast information in a combined MRI+PA imaging can be beneficial.^279^ Previously, a dual-modal PA/MRI probe was designed by doping magnetic ${\mathrm{Ni}}^{2+}$ ions into CuS NPs.^180^ *In vivo* dual imaging of the lymphatic system of a mouse was done. Before injection of the agents, LNs were not visible as there was nearly no signal from the LN in either the PA or MRI images. However, after the contrast agent injection, the LN was clearly visible with significantly enhanced PA and MRI signals from the nodes. In another work, based on Prussian blue, an activatable PA/MRI probe was developed for ${\mathrm{ONOO}}^{-}$ imaging.^278^ In the presence of ${\mathrm{ONOO}}^{-}$, ${\mathrm{Fe}}^{\mathrm{II}}$ within Prussian blue was oxidized into ${\mathrm{Fe}}^{\text{III}}$, resulting in the breakage of the crystal structure of Prussian blue. The changes of probe structure resulted in a decrease in PA signal at 710 nm and activation of MRI signal. The selective activation made the probe promising for monitoring ${\mathrm{ONOO}}^{-}$ accurately deep in the tissue. The core-shell structure nanoprobe was further developed for ratiometric PAI of ${\mathrm{ONOO}}^{-}$, with CuS as the core and PB as the shell. Mice were injected with the nanoprobe and then treated with saline or paracetamol (APAP), which could cause liver damage and lead to producing ${\mathrm{ONOO}}^{-}$. The PA images and MR images of before and 3 h after the injection of APAP are shown in Figs. 11(c) and 11(d), respectively. After the injection of APAP, the liver region exhibited lower PA signals, in contrast to no significant PA signal changes in the saline-injected group. As indicated in Fig. 11(d), the liver region injected with APAP was brighter than that before injection, whereas no obvious changes were found between before and after the saline injection. These outcomes suggested that this dual nanoprobe could be used as an effective tool for detecting how ${\mathrm{ONOO}}^{-}$ changed during drug-induced hepatotoxicity. In addition, lipid-micelles, incorporated with SP dots and a photosensitizer, were designed for dual-modal PA/MRI imaging-guided PTT/PDT.^259^ The prepared nanomaterials exhibited good water dispersibility and low cytoxicity.

### 6.4 Photoacoustic-X Ray Computed Tomography Probes

PEGylated ${\mathrm{TiO}}_{2}$ doped with W (WTO) NPs were developed via an organic route for dual-modal PA+CT imaging.^280^ These WTO NPs not only exhibited strong light absorption in NIR-II region but also absorbed x-rays. Thus, they were a good candidate for a dual-modal CT/PA imaging contrast agent. The effectiveness of WTO NPs in tumor imaging was shown by increased CT contrast and PA signals in the tumor region after contrast agent injection. Another nanocomposite of gold core and silver shell was reported as a dual-modal agent for enhancing contrast in both PA and CT.^281^ Via surface modification with the amphiphilic deblock polymer, the nanocomposite was then functionalized with transferrin for targeting the transferrin receptor. The specific binding to the tumor cells, which overexpressed the transferrin receptor, was shown by combined CT/PA imaging. In addition, based on PEGylated ${\mathrm{WS}}_{2}$ nanosheets, a novel generation of a photothermal agent was developed as a CT/PA imaging probe for tumor imaging.^282^ Au nanostars capped by albumin were also presented as dual-modal PA/CT contrast agents due to their strong optical absorption, good biocompatibility, and good x-ray attenuation.^167^

### 6.5 Photoacoustic-OCT Probes

A common feature of both OCT and PAI is the use of a light source to illuminate the sample but varied detection, optical for OCT and acoustic for PAI. For OCT, the penetration depth is limited to a few millimeters whereas the spatial resolution can reach few microns range. On the other hand, PAI can achieve deep tissue imaging at a few centimeters, but it has a lower spatial resolution than OCT. To overcome the penetration depth-resolution duality, a combination OCT+PAI system was reported.^283^ For example, contrast-enhanced dual-modal OCT/PAM for evaluating retinal diseases was shown with gold nanostars.^166^ Functionalized with an RGD peptide, the gold nanostars exhibited strong optical absorption in the NIR window and excellent photostability, leading to visualization of choroidal neovascularization. Polymeric micelle, due to its small-size structure, was one type of commonly used NP for drug delivery, but the slow drug release rate hindered its translation to the clinic.^267^ To overcome this problem, redox-responsive polymeric micelles nanocarriers were developed. These nanocarriers exhibited efficient response in tumor cells and quick release of the drug, indicating its potential as a theranostic agent for OCT/PA imaging guided chemotherapy.

### 6.6 Photoacoustic-Raman Probes

The goal of increasing specificity can also be achieved using a complementary technique that is more specific but may have other drawbacks. One such example is Raman spectroscopy (RS), which is able to provide molecular information at the cost of extremely weak signal in which 1 in $10^{6}$ to $10^{8}\text{ photons}$ undergo Raman scattering.^284^ As RS is a purely optical technique, it has a relatively low penetration depth limited by the optical diffraction limit, and the signal is typically acquired using a continuous-wave laser. It can, however, be easily combined with PAI. As such, the use of a multimodal contrast agent that can enhance Raman scattering as well as PA signals would greatly increase the amount of molecular information extractable from an acquisition. A hybrid SERS and raster-scanning optoacoustic mesoscopy probe using CNT coupled with AuNPs on silica microspheres was recently developed and tested on *ex vivo* mouse brain tissue.^285^ This probe was operated using 532 nm for the PAI and 785 nm for the Raman imaging. It was also successfully used for 1064-nm PA flow cytometry with increased contrast. Another NIR-II probe based on Au@Au–Ag dot-in-cubic nanoframes (DCFs) was recently demonstrated to have tunable properties across 700 to 1400 nm.^286^ This probe (NIR-II plasNF) was modified with IR-1061 dye-encoding, PEGylation, and cyclic Arg-Gly-Asp (RGD) peptide and then deployed for *in vivo* tumor imaging of mice injected with U87-MG cell clusters, using SERS at 1064 nm up to 4 mm in depth as well as PAI at 995 nm.

Analogous to endogenous PA contrast, biological tissue usually appears with characteristic peaks at Raman shifts of roughly 700 to $1800\;{\mathrm{cm}}^{-1}$, whereas the 1800 to $2800\;{\mathrm{cm}}^{-1}$ is generally silent. This latter silent region can be taken advantage of when synthesizing multimodal probes as demonstrated by Chen et al.,^287^ who developed a probe that had a characteristic peak around $2190\;{\mathrm{cm}}^{-1}$. This probe, a black phosphorus quantum dot-Au hybrid (Au-BPQD nanohybrid), was modified for size with smaller particles (S-ABPs) giving greater SERS signals and larger particles (L-ABPs) having greater NIR absorption for enhanced PA signals. 3D PAI using L-ABPs was demonstrated *in vivo* on 4T1 tumor-bearing BALB/c mice at 680, 808, and 1064 nm excitation with clear improvements in NIR absorption. L-ABPs were also used for PTT and were effective in stopping tumor growth with 3 min of exposure. SERS imaging using S-ABPs was demonstrated *in vivo* on zebrafish embryos.

### 6.7 Triple-Modal Probes

Triple-modal imaging systems were also developed to combine the advantages of more than two modalities and overcome the inherent drawbacks of each individual modality. Dual-modal PA and MR imaging was combined with fluorescence imaging,^288^ PET,^206^ SERS,^266^ and US imaging ^289^ for triple-modal imaging. IR825, which is an analog of ICG, was encapsulated into C18PMH-PEG-Ce6 for a multifunctional contrast agent for PA, fluorescence, and MRI.^288^ In addition to tumor targeting, tumor cell ablation can also be integrated with the combination of PTT and PDT in triple-modal imaging. Fan et al. developed an MNP, which can be agent of not only PAI but also PET and MRI.^206^ The further conjugation with $\alpha_{v}\beta_{3}$ enabled targeted tumor imaging. The multimodal properties of MNP made it promising for molecular theranostics and translation to the clinic. It is difficult to obtain PA images through the scalp and the skull, limiting the application of PAI in brain tumors. To address this problem, triple-modal MRI/PA/SERS NPs were synthesized, allowing for effective accumulation in the tumor region.^266^ Through the intact skull, the tumor delineation was achieved in all three modalities, verifying the potential of the NPs for more accurate brain tumor imaging and resection. Another nanomaterial for PA/US/MR imaging, Prussian blue nanocube (PBNC), was proposed by Kubelick et al.^289^ PBNC can be employed for guiding stem cell injection during the operation, as well as monitoring stem cell therapies after the operation. US/PA images were obtained when PBNC-labeled stem cells were injected into the spinal cord, whereas US/PA/MR images were acquired after the surgery.

## 7 Theranostic Probes

Photo-induced therapy, such as PTT and PDT, uses light-based techniques to kill cancer cells. PTT utilizes agents that have high absorbance in the NIR region to generate heat, resulting in thermal ablation of target cells. PDT employs photosensitizers to transfer laser energy to the surrounding oxygen molecules, leading to the generation of reactive oxygen species to kill the cells. Recently, image-guided therapy has shown great prospects for cancer treatment by offering the following essential information such as (a) tumor size, location, and the relationship between tumor and surrounding tissue; (b) optimal time when the agent reaches the highest level in the targeted region; and (c) the progress of disease after therapy.^288^ Among different imaging modalities, PAI provides the advantages of strong contrast, deeper penetration than pure optical imaging, and scalable spatial resolution, making it a competitive imaging method for guiding therapy.

PAI-guided PTT has previously been proposed for the imaging and alleviation of pain^171^ as well as imaging and ablation of tumors.^106^^,^^202^^–^^204^^,^^227^^,^^260^^,^^290^ For example, ICG-loaded polydopamine-iron ions coordination nanoparticles (PDA-FE3+-ICG NPs) were reported as PA and MR dual-modal agents, which passively accumulate in the tumor via an EPR effect.^260^ Effective PTT treatments with low laser density were achieved with efficient ablation of the tumor and minimal side effects. PAI-guided PDT has also been explored. Lin et al.^291^ reported two-dimensional Te nanosheets that were synthesized via a facile liquid exfoliation technique. Under laser illumination, high PAI performance and adequate ROS production for PDT was shown. Ding et al.^292^ demonstrated that nanodots modified by Pluronic $F_{127}$ and folic acid can produce singlet oxygen under an 808-nm laser, which was widely employed for PDT of cancer. The strong absorption at 808 nm also made the nanodots competitive contrast agents for PAI.

**Fig. 12:**
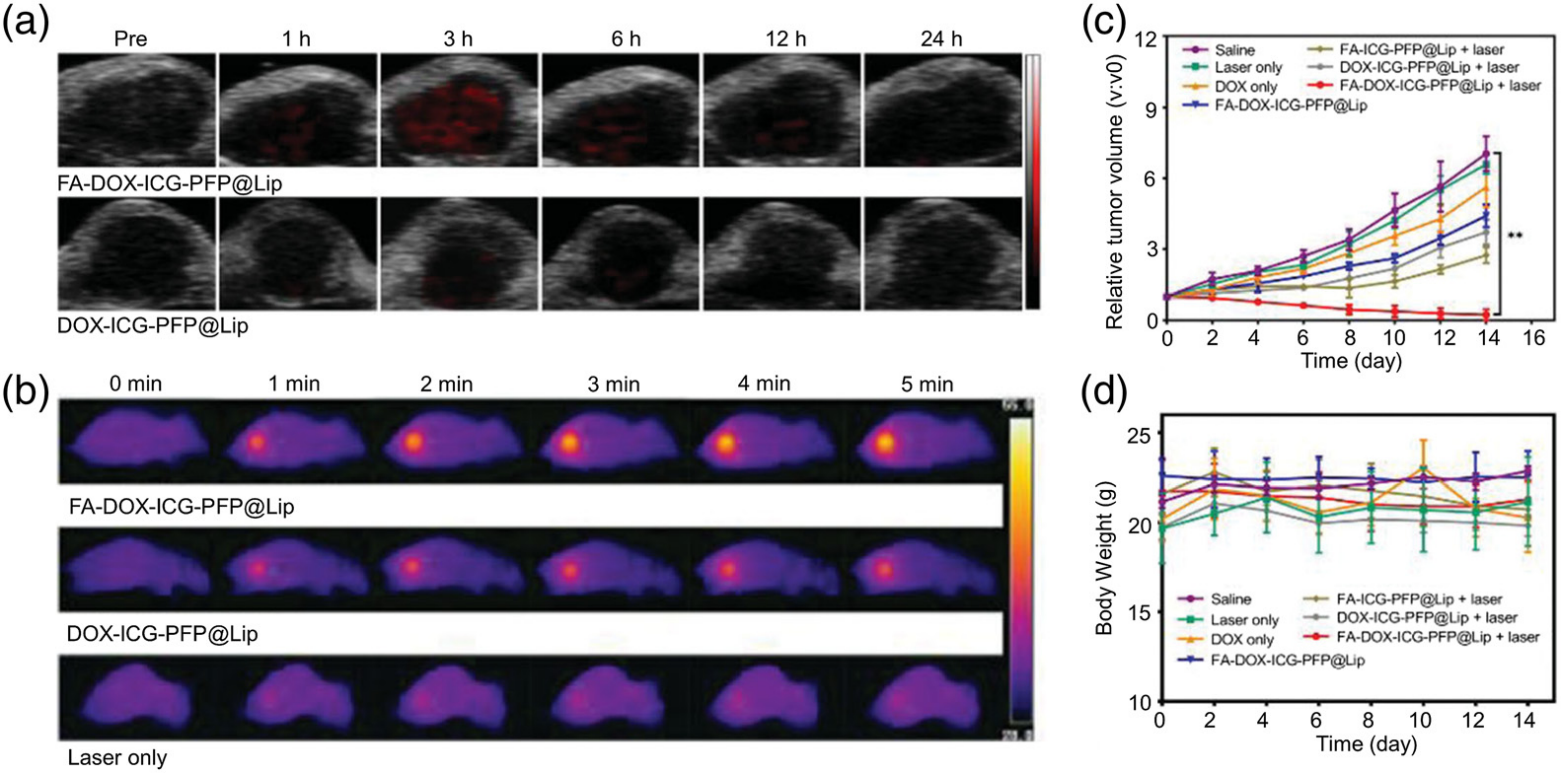
(a) PA images of tumors in tumor-bearing mice before and after injection of FA-DOX-ICG-PFP@Lip or DOX-ICG-PFP@Lip. (b) Thermal images of tumor-bearing mice injected with FA-DOX-ICG-PFP@Lip, DOX-ICG-PFP@Lip and saline under 808-nm laser irradiation ($1\;W/{\mathrm{cm}}^{2}$, 5 min). (c) Relative tumor volume changes of seven groups of tumor-bearing mice. (d) Body weights of seven groups of mice during the 14-day period. Reprinted with permission from Ref. 101.

However, the unsatisfactory results of a single therapeutic treatment prompted attention to the development of combination therapy, such as PTT and chemotherapy,^101^^,^^262^^,^^293^ PDT and chemotherapy,^294^ and PTT and PDT.^258^^,^^259^^,^^288^^,^^295^ FA-DOX-ICG-PFP@Lip was proposed as a laser-triggered contrast agent that targets retinoblastoma and, moreover, ablates tumor and helps drug delivery under laser excitation.^101^ As a folate-receptor targeted laser-activatable liposome, FA-DOX-ICG-PFP@Lip was a PA/US dual-modal contrast agent, loaded with doxorubicin, ICG, and liquid perfluoropentane. It could also be used in imaging-guided chemo/photothermal retinoblastoma (RB) therapy. As the folic acid receptor is overexpressed in RB, the NPs coated with folic acid can target the tumor site. Liposomes are commonly used for the delivery of antitumor nanomedicine and DOX is a chemotherapeutic antitumor medicine. ICG and PFP are contrast agents for PAI and US imaging, respectively. Except for enhancing contrast in US imaging, liquid PFP vaporizes to microbubbles by optical droplet vaporization, which can augment cell permeability and enhance drug release, namely, DOX release. FA-DOX-ICG-PFP@Lip binds to FRs on RB cells and accumulates to due EPR. Both *in vitro* and *in vivo* capabilities of FA-DOX-ICG-PFP@Lip in PA/US imaging were evaluated in the agar gel model and tumor-bearing mice, respectively. As for the *in vivo* experiment, FA-DOX-ICG-PFP@Lip showed a stronger PA signal and longer retention than the DOX-ICG-PFP@Lip group, indicating its effective accumulation in tumor sites [Fig. 12(a)]. The photothermal capability of FA-DOX-ICG-PFP@Lip was evaluated *in vitro*. When irradiated with an 808 nm laser, FA-DOX-ICG-PFP@Lip converted light energy to heat, causing an immediate temperature rise to more than 42°C (hyperthermia threshold) and thus destruction of tumor cells, meanwhile, PFP phase transformation was initiated to release DOX, resulting in both photothermal and chemotherapeutic antitumor effects. The synergistic effect of FA-DOX-ICG-PFP@Lip was also evaluated *in vivo*. FA-DOX-ICG-PFP@Lip, DOX-ICG-PFP@Lip, and saline were injected into the mice through tail vein, and an 808-nm laser was employed to irradiate the tumor region. A thermal imaging camera was used to observe the photothermal conversion efficiency during 5-min laser irradiation. As shown in Fig. 12(b), the FA-DOX-ICG-PFP@Lip group exhibited a significant temperature increase and reached 50.1°C after 5 min, whereas the DOX-ICG-PFP@Lip group showed a smaller temperature rise to only 42.5°C, and the control group temperature remained nearly unchanged. Tumor-bearing mice were divided into seven groups: saline, saline + laser, DOX solution, FA-DOX-ICG-PFP@Lip, FA-DOX-PFP@Lip+laser, DOX-ICG-PFP@Lip+laser, and FA-DOX-ICG-PFP@Lip+laser. In the FA-DOX-ICG-PFP@Lip group, tumor shrunk gradually and disappeared after 14-day treatment, whereas tumor did not show an obvious decrease in other groups [Fig. 12(c)]. Figure 12(d) presented that there was no significant change in the body weight of the mice over the whole treatment period, indicating the potential of FA-DOX-ICG-PFP@Lip and laser for tumor treatment.

In addition, P-hyd-IM was reported as a fluorescence/PA dual-modal self-targeting contrast agent that integrates ICG with methotrexate (MTX) as a chemotherapeutic drug, followed by surface functionalization with acidity-responsive PEG.^262^ PEG can prolong circulation time due to self-targeting function shielding in blood and enhances cellular uptake for MTX due to the activation trigger in the tumor site. When P-hyd-IM was delivered to acidic tumor tissue due to passive targeting, PEG responded to the acid microenvironment and was separated from the ICG-MTX (IM) core to expose MTX ligands to activate the self-targeting of IM. This resulted in the rapid release of MTX for chemotherapy via dual stimulation of both inner acidity and external laser excitation. Fluorescence imaging/PAI was employed to monitor the tumor elimination under 808-nm laser excitation. *In vitro* photothermal and PA properties were also shown. P-hyd-IM showed quicker temperature changes, higher maximum temperature, and the same temperature rise during five cycles of laser exposure compared with the IM and ICG groups, indicating that P-hyd-IM possessed high photothermal conversion efficiency and photothermal stability. In addition, *in vivo* fluorescence imaging and PAI were conducted in HeLa tumor-bearing mice with an 808 nm laser, showing stronger fluorescence and PA signals and longer retention time and indicating effective tumor accumulation capability. In addition, the antitumor efficacy was assessed *in vivo*. HeLa tumor-bearing mice were injected with P-hyd-IM, and the tumor sites were irradiated under an 808 nm laser. The combination of P-hyd-IM and laser irradiation showed high inhibition efficiency on tumor growth, as evident from the complete elimination of the tumor.

Compared with standalone PTT or PDT, the synergistic combination of PTT and PDT can show enhanced anticancer efficiency.^265^^,^^295^ PPy has the advantage of strong optical absorption in the NIR region, photothermal stability, and biocompatibility. MB conjugated with PPy (PPy-MB NPs) was designed as an NIR photoabsorber to achieve PAI-guided combined PTT/PDT therapy.^295^ Once irradiated with the 880-nm laser, PPy-MB NPs not only generated a large amount of ROS and increased the temperature rapidly to kill cancer cells but also showed a strong PA signal even at a low concentration ($63\;\mu g\;{\mathrm{ml}}^{-1}$) in the phantom test. In another work, human serum albumin (HSA)-ICG nanoparticles (HSA-ICG NPs) were designed via intermolecular disulfide conjugations, which presented effective accumulation in tumor and long retention time in 4T1 tumor-bearing mice.^265^ Fluorescence and PA dual-modal imaging were conducted to show high contrast between tumor and normal tissues. Meanwhile, the NPs can also induce ROS and local temperature rise for both PDT and PTT treatments. These results suggest that HSA-ICG NPs have potential for dual-modal imaging-guided synergetic phototherapy. Imaging-guided PDT and chemotherapy were shown in this work.^294^ Gelatin nanogel core loaded with MB and cisplatin (MPNGs), followed by fusion with RBC vesicles (MPV), was developed. Via PA and fluorescence imaging, MPV showed precise tumor targeting in 4T1 tumor-bearing mice. Meanwhile, under laser irradiation, the MPV generated hyperthermia and released MB and cisplatin, effectively killing breast cancer cells. The results illustrated that MPV can be used as a promising contrast agent for triple-modal imaging-guided PDT/ chemotherapy.

Finally, a combination of PDT and adipose browning induction under PA molecular imaging guidance was developed for the reversal of diabetes.^296^ Although PDT can induce localized and immediate cell apoptosis, it is restricted by optical scattering at larger depths. Browning can convert white adipocytes, which store energy, into brown-like adipocytes, which dissipate energy as heat, as a means of reducing obesity. However, this takes place at a longer timescale than PDT. Thus, the $P_{\mathrm{at}}\text{-}\mathrm{HBc}/\mathrm{RSG}\&{\mathrm{ZnPcS}}_{4}$ probe used zinc phthalocyanine tetrasulfonate (${\mathrm{ZnPcS}}_{4}$) as a photosensitizer and PA agent, rosiglitazone (RSG) as the PPARγ agonist to convert white adipose tissue into its thermogenic counterpart, and Hepatitis B core (HBc) for the biocompatible delivery of the probe as well as the adipose-targeting peptide motif ($P_{\mathrm{at}}$) for prohibitin binding. The combinatory probe provided effective tracking using PA *in vivo* in Ob/Ob mice as it accumulated in greater quantities in the different types of white adipose tissue compared with nontargeted accumulation. It was also noted that the better the blood flow to the tissue was, the greater the targeting effect was. The fat-reduction capabilities were also demonstrated *in vivo* in which mice that received two cycles of the PDT-browning treatment (20 min of $50\;\mathrm{mW}/{\mathrm{cm}}^{2}$) lost 10% of their weight compared with groups on the same diet but given either only $P_{\mathrm{at}}\text{-}\mathrm{HBc}/{\mathrm{ZnPcS}}_{4}$ or only $P_{\mathrm{at}}\text{-}\mathrm{HBc}/\mathrm{RSG}$. Throughout the trial, PAI was used to monitor changes such as angiogenesis.

## 8 Discussion

Label-free PAI can provide high-resolution, deep tissue imaging. Compared with pure optical imaging, hybrid PAI offers several advantages and is making good progress for various preclinical and clinical applications.^297^^,^^298^ Without using any contrast agents, PAI provides optical absorption-based contrast images mainly utilizing the intrinsic contrast coming from chromophores, such as blood and melanin. However, the use of PAI will be limited if we only depend on the intrinsic contrast. Hence, the use of contrast agents or PA probes opens avenues for a wide range of applications in addition to improving the imaging depth.^86^ As of January 2021, Seno Medical’s breast cancer diagnostic technology (based on the combination of ultrasound and PA)^299^ is the first FDA-approved PA device for clinical application. As time goes by, more and more applications are expected to get FDA approval for using PA technology. Hence, developing high-performance PA probes for accurate and sensitive *in vivo* diagnosis of complicated and deeply located diseases is very important.

Table 1 lists some of the most recent probes developed for PAI according to their material composition. In addition, the peak absorption wavelengths of the probes, targets for molecular imaging, conjugates, and intended application are also listed where available. It should be noted that absorbance is often broadband, and the peak absorption wavelength may not be utilized in favor of increased side band absorption that often overlaps into the NIR-I or NIR-II ranges. Although probes may exhibit excellent absorption at a certain wavelength, they may be used at an entirely different wavelength (typically in NIR) for PAI. A second factor for this choice is the difference between the reference spectrum and the probe’s absorbance spectrum. If the difference of a side band is more/easily differentiable from endogenous or nonactivated signals compared with the peak wavelength, it can provide better contrast. For dual-modal fluorescence imaging probes, this peak absorption wavelength does not need to be NIR to begin with as the emission often spans NIR-II or can be tuned.

**Table 1:** List of exogenous PA contrast agents sorted by material, target, and application.

Probe abbreviation	Target or conjugate	Peak absorption wavelength	Intended/demonstrated application	Additional attributes
**Metallic NPs**				
OVA-GC-AuNPs^247^	Tumor via EPR	538 nm	Metastatic LN imaging	—
AgI/AuNRs^158^	RONS	780 nm	RONS sensing	—
c(RGDyk)-MHDA/LSC@AuNP^153^	pH	680 nm	Tumor imaging	—
GC-AuNPs^156^	Tumor via EPR	528 nm	Tumor imaging	—
MAPS^155^	EGFR	515, 650 nm	Cancer cell imaging	—
Miniature AuNRs^159^	GRPR	1064 nm	Prostate cancer imaging	—
W-VO2@PEG^229^	Temperature	NIR-II	Tumor imaging	Phototherapy
PB nanoprobe^278^	${\mathrm{ONOO}}^{-}$	710 nm	Drug-induced liver injury	Activatable, MRI + PAI
Au@Au–Ag DCFs^286^	Integrin $\alpha_{v}\beta_{3}$	1138 nm	Tumor imaging	SERS + PAI
GNSs^166^	Integrin $\alpha_{v}\beta_{3}$	650 nm	Choroidal neovascularization	OCT + PAI
MoS2-AuNRs^171^	NGF	710 nm	OA pain imaging	Phototherapy
Au-RRVR^154^	Furin enzyme, pH	NIR	Tumor imaging, therapy	Phototherapy
FA-ZnPcNDs^292^	${\mathrm{PEG}}_{2000}$-folate, EPR	808 nm	Tumor imaging, therapy	Phototherapy
$P_{\mathrm{at}}\text{-}\mathrm{HBc}/\mathrm{RSG}\&{\mathrm{ZnPcS}}_{4}$ ^296^	Prohibitin–adipose tissue	625 nm	White adipose tissue (fat) imaging, therapy	PDT and browning
GNR@PDAs^170^	Nontargeted	512, 1090 nm	High contrast imaging	—
NiPNP^181^	Nontargeted	1064 nm	Tumor imaging	—
M-AuHNRs^175^	Nontargeted	700 nm	Tumor imaging	—
TiN NP^283^	Nontargeted	NIR	Nanodentistry	OCT + PAI
CNT-AuNP-coated silica microspheres^285^	Nontargeted	561 nm	Brain imaging	SERS + PAI
Au-BPQD nanohybrids^287^	Nontargeted	546 nm	Tumor imaging and therapy	SERS + PAI, phototherapy
mTiO2@PPY-HNK^276^	Nontargeted	UV	Tumor imaging	US + PAI, chemotherapy
PEGylated WTO NPs^280^	Nontargeted	NIR	Tumor imaging, therapy	CT + PAI, phototherapy
Mg-based micromotors^255^	—	750 nm	GI tract imaging, therapy	—
Nickel-based spherical Janus microrobots^182^	Nontargeted	780 nm	Vasculature imaging	—
**Small organic molecules**				
Cypate-dimer^199^	LS301	778 nm	Tumor imaging	—
800RS-PMPC^192^	Tumor via EPR	771 nm	Colon cancer imaging	—
SDKNP^257^	$K^{+}$	NIR	Tumor imaging	—
B7-H3-ICG^172^	B7-H3 (CD276)	NIR	DCIS imaging	US, fluorescence, PAI
Pan800^188^	EGFR	710, 770 nm	Metastatic LN imaging	Fluorescence, PAI
CRANAD-2^250^	Brain Aβ deposits	NIR	Brain imaging	Fluorescence, vMSOT
FA-DOX-ICG-PFP@Lip^101^	FR	800 nm	Retinoblastoma imaging, therapy	US + PAI, photo/chemotherapy
IM^262^	pH	812 nm	Tumor imaging, therapy	Photo/chemotherapy
ICG/LAP-PDA-PEG-RGD/DOX nanoplatforms^293^	Integrin $\alpha_{v}\beta_{3}$	485 nm	Tumor imaging	Photo/chemotherapy
**Organic NPs**				
cRGD-MNPs^248^	Integrin $\alpha_{v}\beta_{3}$	—	Tumor imaging	—
SBC-EV(ICG/PTX)^201^	pH	780 nm	Tumor imaging, therapy	SDT
Mito-BDP5^208^	Mitochondria via EPR	648 nm	Tumor imaging	Fluorescence, PAI
OTTAB NPs^231^	NO	690 nm	Encephalitis detection	Activatable
QCs^207^	MMP-2	619 nm	Tumor imaging	Activatable
PBNCs^289^	Stem cells	734 nm	Stem cell imaging and monitoring	US + MRI + PAI
Por−DPP NPs^202^	Tumor via EPR	745, 807 nm	Tumor imaging, therapy	Phototherapy
NIR-II RGD-conjugated BTB NPs^270^	Integrin $\alpha_{v}\beta_{3}$	730 nm	Tumor, vasculature imaging	Fluorescence, PAI
SDSP micelles^267^	GSH	271 nm	Tumor imaging, therapy, vasculature imaging	OCT + PAI, chemotherapy
DRM NPs^290^	Nontargeted	790 nm	Tumor imaging, therapy	Phototherapy
**Semiconducting polymer NPs**				
BTNPs^214^	Macrophages	854 nm	Inflammation imaging	—
RSPN^216^	${O_{2}}^{•-}$	690, 800 nm	Atherosclerosis imaging	—
SPNP^104^	Granzyme B	700, 760 nm	Cytotoxic T cell imaging	Activatable, fluorescence, PAI
MSPN^232^	pH	535, 780 nm	Tumor imaging	Activatable, fluorescence, PAI
SPN@RBCM^106^	Tumor via EPR	840 nm	Tumor imaging, therapy	Phototherapy
SPNs^212^	Non targeted	929, 1030 nm	Brain tumor imaging, therapy	Phototherapy
SSS-micelles^211^	Tumor via EPR	1255 nm	Tumor imaging, therapy	phototherapy
**Proteins**				
miRFP670-iRFP720 FRET biosensor^223^	Caspase-3	NIR	Cell apoptosis imaging	Activatable
mDrBphP-PCMm/F469W^239^	Tumor via *E. coli* MG1655	780↔630 nm	Tumor imaging	Photoswitching
rsGCaMP1.1 & 1.4-er^224^	${\mathrm{Ca}}^{2+}$	405, 488 nm	Calcium imaging	Photoswitching
GCaMP6f^74^	${\mathrm{Ca}}^{2+}$	488 nm	Calcium imaging	—
iGECI^253^	${\mathrm{Ca}}^{2+}$	640, 700 nm	Calcium, brain imaging	Fluorescence, PAI
ReBphP-PCM, RpBphP1-PCM^238^	Non targeted	770↔680 nm	T cells, bacteria, tumor imaging	Photoswitching
**Inorganic particles and others**				
DPA voltage sensor^256^	Plasma membrane	—	Brain voltage response imaging	—
SWNTs^217^	$\mathrm{Ly}\text{-}6C^{\mathrm{hi}}$ monocytes	NIR	Inflamed atherosclerotic plaque imaging	—
DOX@CNH-PG-Au^222^	Tumor via EPR	530 nm	Tumor imaging, therapy	Radiochemotherapy
PPC NPs^242^	Temperature	NIR	Tumor imaging	Fluorescence, PAI
5K-HA-HPPS^249^	CD44 and SR-B1	748 nm	Metastatic LN imaging	Fluorescence, PAI
PBB5^251^	Brain tau deposits	590 to 690 nm	Brain imaging	Fluorescence, vMSOT
CPZ III^254^	${\mathrm{Ca}}^{2+}$, pH	610, 660 nm	Calcium, tumor imaging	—
DYE-VEGF-antibody-loaded microbubbles^275^	VEGF	—	Inflammatory arthiritis	Contrast-enhanced US + PAI
DATN^227^	NO, pH	680, 950 nm	Tumor imaging, therapy	Activatable, phototherapy
PAPSI^240^	Nontargeted	680↔980 nm	Cancer cell imaging	Photoswitching
DCNP@PDA NP^263^	Nontargeted	NIR	Gastrointenstinal tract imaging	Fluorescence, PAI

Abbreviations: RONS, reactive oxygen and nitrogen species; EGFR, epidermal growth factor receptor; NGF, nerve growth factor; GRPR, gastrin-releasing peptide receptor; VEGF, vascular epithelial growth factor; GSH, glutathione; FR, folic acid receptor; EPR, enhanced permeability and retention; NIR, near-infrared; PA, photoacoustic; vMSOT, volumetric multispectral optoacoustic tomography; OCT, optical coherence tomography; SERS, surface-enhanced Raman scattering; SDT,- sonodynamic therapy; US, ultrasound imaging; MRI, magnetic resonance imaging.

The materials used for constructing the probe can mainly be classified as metallic, organic small molecules/ dyes, organic NPs, SPs, inorganics, or others. Depending on how they interact inside the body, they can be divided into either nontargeted or targeted contrast agents. According to the wavelength of laser excitation to which they respond, the PA agents can be divided into NIR-I or NIR-II contrast agents. Finally, depending on how the PA signal is altered, they can either be considered always on or “activatable” contrast agents. We first discussed the recently proposed contrast agents from the perspective of material. Considering the safety issues, more and more organic NPs are being designed (e.g., via dye encapsulation) due to low toxicity and biodegradability, especially the SP NPs, which can also be synthesized as activatable probes. Despite being composed of metals, metallic NPs still show great promise due to strong optical absorbance, higher photothermal conversion efficiency, and photostability. The copper-based and gold-based NPs will attract great attention due to having tunable absorption peaks into the NIR-II window, which allow for deep imaging. However, cytotoxicity and cellular uptake studies should be stressed to evaluate their effect on the human body.

In addition to materials and conjugations for targeting, an increasing emphasis is being put on the development of probes that allow for greater control once injected. Thus, the use of activatable and controllable probes provides an especially promising prospect for PAI to reach greater depths with greater control. The main advantage of these probes stems from the high-contrast differential images of a region before and after activation. Biomarker-activatable probes enable specific imaging of deep-seated tumors or trace amounts of early indicators wherever they may be. Photoswitching probes that are now able to bind to targeted receptors allow this process to become rapidly reversible using varying excitation wavelengths for the use of lock-in detection. Temperature-dependent probes take advantage of localized changes in heat that can be activated externally and are also progressing toward reversible modulation. These highly specific probes have all been deployed for various *in vivo* applications, such as SNL detection, brain disease characterization, cancer, tumor microenvironment imaging, atherosclerosis characterization, and more. Although PAI in the early days suffered from low contrast or nonspecific contrast, molecular agents are being tailored more and more for each specific disease and its most optimal targeting.

Another promising direction of interest is the multimodality of contrast agents. Such probes can take advantage of other imaging modalities to aid PAI and achieve better quality images with greater penetration depth. These include the higher intensity of fluorescence signals, the intrinsic molecular information of RS, the wider fields of view from MRI, and others. Several probes have been developed to take advantage of NIRF and PAI as they can utilize overlapping fluorescent dyes. US imaging and PAI have also been combined due to their ease of integration using the same receiving electronics but with the added benefit of much deeper visualization. Probes used in both US and PA, such as microbubbles, can be used to facilitate simultaneous SDT or phototherapy and imaging. Other probes facilitate dual-mode enhancement in modalities that already use contrast, such as MRI, CT, and SERS. Certain probes even allow PAI to be coupled with optical interferometric techniques such as OCT and enhance signals for both modalities. The ability to retain the same agent for dual or triple modality imaging provides a truly promising prospect for complete diagnoses within a single imaging session. Like multimodal imaging, these targeted probes have been used to facilitate not only the imaging of a specific tumor site but also the therapy. This is particularly advantageous as PDT, PTT, and SDT are already being utilized for the treatment of tumors. Numerous probes that are able to facilitate thermal ablation or deploy drugs after localizing within the target without the need for secondary contrasts have been developed. This synergy is not limited to cancer therapy as fat reduction using dual-modal phototherapy and browning was also recently demonstrated.

The ongoing research of molecular PAI is progressing in several promising directions such as activatable and multimodal probes. However, it is critical to note that the translation of such research into the clinic often requires extensive regulatory approval due to biosafety and biocompatibility issues. Though many probes incorporate this aspect into their development, it requires active pursuit if these exciting probes are to come to market and have a significant impact on patient diagnoses.

## 9 Conclusion

This review aims to present a comprehensive view of PA molecular optical probes for various applications in recent years. With the aid of contrast agents, PAI can provide high-resolution *in vivo* imaging of deep-seated targets. With specific targeting, molecular information can also be obtained. With multimodal imaging/theranostic probes, image-guided treatment of diseases will be a key development in the future. Despite all of the positive aspects of contrast agent-enhanced PAI, effort must still be put into making biocompatible, safe agents. This will ensure regulatory clearances are easily obtained for these probes to be used clinically and to have a real impact on patient lives.
